# Solar energy storage at an atomically defined organic-oxide hybrid interface

**DOI:** 10.1038/s41467-019-10263-4

**Published:** 2019-06-03

**Authors:** Christian Schuschke, Chantal Hohner, Martyn Jevric, Anne Ugleholdt Petersen, Zhihang Wang, Matthias Schwarz, Miroslav Kettner, Fabian Waidhas, Lukas Fromm, Christopher J. Sumby, Andreas Görling, Olaf Brummel, Kasper Moth-Poulsen, Jörg Libuda

**Affiliations:** 1 Lehrstuhl für Physikalische Chemie II, Friedrich-Alexander-Universität Erlangen-Nürnberg, Egerlandstr. 3, 91058 Erlangen, Germany; 2 Department of Chemistry and Chemical Engineering, Chalmers University of Technology, 41296 Gothenburg, Sweden; 3 Lehrstuhl für Theoretische Chemie, Friedrich-Alexander-Universität Erlangen-Nürnberg, Egerlandstr. 3, 91058 Erlangen, Germany; 4 Department of Chemistry and the Centre for Advanced Nanomaterials, The University of Adelaide, Adelaide, South Australia 5005 Australia; 5 Erlangen Catalysis Resource Center, Friedrich-Alexander-Universität Erlangen-Nürnberg, Egerlandstr. 3, 91058 Erlangen, Germany

**Keywords:** Energy, Light harvesting, Surface assembly, Surface spectroscopy

## Abstract

Molecular photoswitches provide an extremely simple solution for solar energy conversion and storage. To convert stored energy to electricity, however, the photoswitch has to be coupled to a semiconducting electrode. In this work, we report on the assembly of an operational solar-energy-storing organic-oxide hybrid interface, which consists of a tailor-made molecular photoswitch and an atomically-defined semiconducting oxide film. The synthesized norbornadiene derivative 2-cyano-3-(4-carboxyphenyl)norbornadiene (CNBD) was anchored to a well-ordered Co_3_O_4_(111) surface by physical vapor deposition in ultrahigh vacuum. Using a photochemical infrared reflection absorption spectroscopy experiment, we demonstrate that the anchored CNBD monolayer remains operational, i.e., can be photo-converted to its energy-rich counterpart 2-cyano-3-(4-carboxyphenyl)quadricyclane (CQC). We show that the activation barrier for energy release remains unaffected by the anchoring reaction and the anchored photoswitch can be charged and discharged with high reversibility. Our atomically-defined solar-energy-storing model interface enables detailed studies of energy conversion processes at organic/oxide hybrid interfaces.

## Abstract (web summary)

Molecular photoswitches provide an extremely simple solution for solar energy conversion and storage. Here, the authors report on the assembly of an operational solar energy-storing organic-oxide hybrid interface, which consists of a tailor-made molecular photoswitch and an atomically-defined semiconducting oxide film.

## Introduction

The development of new technologies for solar-energy conversion and storage is among the grand challenges in our transition to a renewable energy system^1,2^. Besides the conventional technologies, alternative chemical methods can provide particularly simple solutions for long-term solar-energy storage. Among these methods is energy storage in molecular photoswitches^3–5^, such as the valence couple norbornadiene (NBD)/quadricyclane (QC)^6–15^. In this storage couple, the energy-lean NBD photoisomerizes via an intermolecular cycloaddition to yield the energy-rich QC. This extremely simple one-photon-one-molecule reaction allows storing up to 89 kJ/mol (0.97 MJ/kg) of chemical energy, an energy density that is comparable with state-of-the-art batteries.

The NBD–QC system has been proposed to hold great potential for solar-energy harvesting and storage; however, it also comes with a number of challenges. Recently, several of these challenges, which were encountered in earlier research, could be successfully addressed and the system attracted renewed attention^12,13^.

One issue is related to the fact that pristine NBD absorbs light at wavelengths below 300 nm only. Therefore, photosensitizers are required to red-shift the absorption^8,16^. These photosensitizers, however, cause stability issues, not only during photoconversion but also during the catalytically triggered energy release. Typically, this problem is tackled by using substituted NBDs that absorb light at much larger wavelength^17,18^. Such modifications, however, come at the price of increasing the molecular weight and, thereby, decreasing the energy density. Some authors of this work recently suggested a variety of compounds that combine beneficial absorption properties and low molecular weight^18^. Alternatively, the energy density can be increased by attaching more than one NBD unit to a single chromophore^19^. A second issue is related to the fact that red-shifting the absorption maximum of NBD often destabilizes the corresponding QC isomer. However, this challenge could also be addressed by molecular design^20,21^. Recently, macroscopic heat release from a molecular solar thermal (MOST) storage device was demonstrated with a reversibility of more than 99.8% per storage cycle^13^. The energy release was triggered catalytically using a carbon-supported cobalt phthalocyanine catalyst.

A particularly fascinating idea is to control the NBD/QC storage system electrochemically^22^. Recently, we have shown that the back conversion can indeed be triggered electrochemically with a reversibility of 95%, even in the presence of an external photosensitizer^23^. The electrochemical approach does not only provide additional control but also holds the potential of converting part of the stored energy directly to electricity^22^. In principle, this may enable the construction of an energy-storing solar cell; however, the NBD unit would have to be coupled to a semiconducting electrode, for instance by attaching it via suitable anchor groups (similar as in a dye-sensitized solar cell).

While anchored NBD films could be prepared recently^24^, a functioning energy-storing hybrid interface, consisting of a photoswitchable NBD monolayer bound to an oxide surface, has not been reported to date. In this work, we demonstrate photochemical switching in an anchored NBD monolayer bound to an atomically defined oxide surface.

**Fig. 1:**
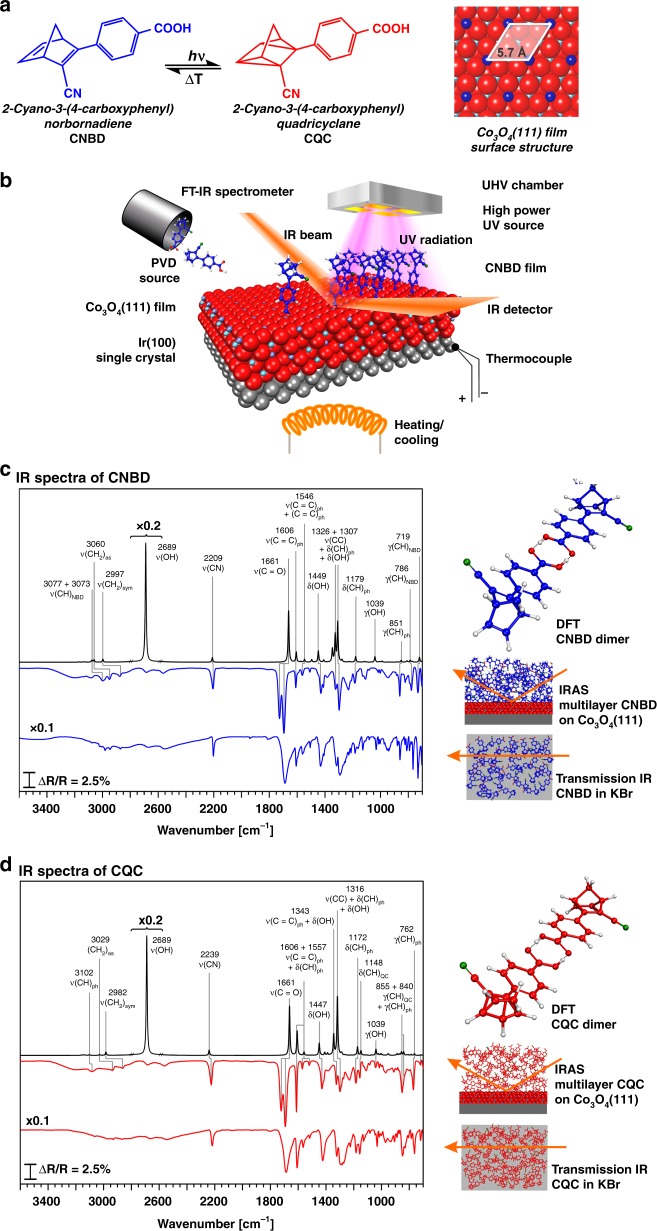
The molecular photoswitch used in this study: **a** molecular structures of the energy-lean isomer CNBD and the energy-rich isomer CQC; **b** schematic representation of the in-situ photochemical IRAS setup in UHV used in this work; **c** IR spectra of CNBD (from top to bottom): IR spectrum calculated by DFT, IR reflection absorption spectrum of CNBD multilayer deposited by PVD referenced to a background of a clean sample, IR transmission spectrum of CNBD in KBr; **d** IR spectra of CQC (from top to bottom): IR spectrum calculated by DFT, IR reflection absorption spectrum of CQC multilayer deposited by PVD referenced to a background of a clean sample, IR transmission spectrum of CQC in KBr

We used a synthesized NBD derivative that features four functionalities (see Fig. 1): (i) an NBD energy-storage unit, (ii) push–pull ligands that red-shift the absorption into the near-visible region, (iii) a spectroscopic marker that allows monitoring the conversion by in-situ IR spectroscopy, and (iv) a carboxylic acid linker group for attachment to the oxide. The NBD derivative is anchored to an atomically defined Co_3_O_4_(111) film with a known surface structure^25,26^. Both the assembly of the hybrid interface and the photochemical energy storage experiments were performed under ultraclean conditions in ultrahigh vacuum (UHV).

Our work demonstrates that it is possible to build an operational solar-energy-storing hybrid interface that consists of a single-anchored NBD monolayer on a well-defined semiconducting oxide surface. The interface is stable and photoswitchable with high reversibility. Our hybrid interface represents a type of model system, in which both the anchored film and the oxide surface are well defined at the atomic level. We believe that the type of model system described herein will enable studies of energy storage and release processes at such interfaces at a high level of detail.

## Results

### Preparation and properties of the model interface

The photoswitchable NBD monolayer was prepared from the NBD derivative 2-cyano-3-(4-carboxyphenyl)norbornadiene (CNBD) shown in Fig. 1a (see the Methods section for synthesis and Supplementary Methods for properties). The molecule comprises four essential functionalities: (i) the NBD storage unit, (ii) the donor–acceptor substituent pair which red-shifts the light absorption region, (iii) the CN group which is used as a marker for IR spectroscopy (see below), and (iv) the carboxylate group which acts as an anchor for oxide surfaces. Upon irradiation, CNBD converts to its energy-rich counterpart 2-cyano-3-(4-carboxyphenyl)quadricyclane (CQC) storing 0.363 MJ/kg in form of chemical energy (see Supplementary Methods). CNBD shows an absorption onset at 378 nm and absorption maximum at 319 nm, while CQC absorbs at much shorter wavelengths (see Supplementary Methods). Therefore, irradiation with ultraviolet (UV) light leads to nearly quantitative conversion of CNBD to CQC.

CNBD films were deposited by physical vapor deposition (PVD) in UHV onto an ordered Co_3_O_4_(111) film (see Fig. 1b). The Co_3_O_4_(111) films (thickness 8 nm) were grown on an Ir(100) single crystal using the procedure introduced by Heinz, Hammer et al.^27^. The surface of the Co_3_O_4_(111) film has been characterized in detail by scanning tunneling microscopy (STM) and low-energy electron diffraction I–V analysis (LEED-IV). It is terminated by a layer of Co^2+^ ions in the tetrahedral positions of the Co_3_O_4_ spinel structure (see Fig. 1a). Note that bulk Co_3_O_4_ is a semiconducting oxide with a bandgap of 1.6 eV^28^, while STM studies suggest that the bandgap is larger for the thin films used in this work^26^. In order to verify that CNBD can be evaporated without decomposition, we deposited a multilayer film onto Co_3_O_4_(111) and compared the infrared reflection absorption spectrum to the spectrum recorded in transmission (see Fig. 1c). Identical bands are found in both spectra, indicating that the deposition of a pure CNBD layer is possible without any decomposition products detected (note that the splitting of the carboxyl stretching band at 1700 cm^−1^ in IRAS indicates dimer formation on the frozen multilayer).

In order to identify the vibrational bands, we performed density functional theory (DFT) calculations of the CNBD dimer and analyzed the corresponding modes (see Supplementary Methods). The experimental and theoretically calculated band positions and their assignments are given in Supplementary Table 1. For this work, the most important feature is the CN stretching band ν_CNBD_(CN) at 2204 cm^−1^ (DFT 2209 cm^−1^). After photoconversion to CQC, the band blue-shifts to 2225 cm^−1^ (ν_CQC_(CN), DFT 2239 cm^−1^). This shift can be clearly resolved by IR spectroscopy, and will be used in this work to follow the interconversion between the two isomers. The complete IR spectra of CQC recorded in reflection and transmission modes and the spectrum calculated by DFT are shown in Fig. 1d. The band positions and assignments are also given in Supplementary Table 1.

### Growth of CNBD films on Co_3_O_4_(111)

**Fig. 2:**
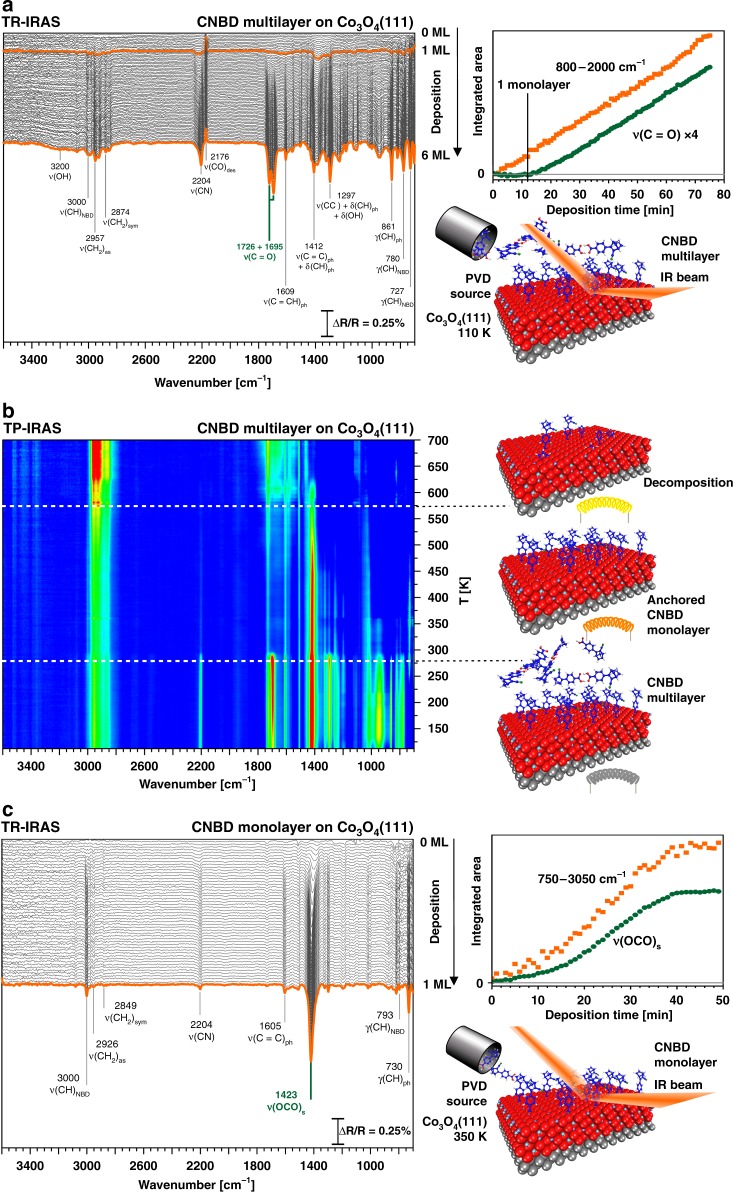
Preparation and stability of CNBD films on an ordered Co_3_O_4_(111) surface: **a** in-situ IRAS during PVD of a CNBD multilayer on Co_3_O_4_(111) at sample temperature 110 K (orange squares: total intensity, green circles: intensity of ν(C = O) band of CNBD); **b** temperature-programmed IRAS after deposition of a CNBD multilayer showing the stability regions of the multilayer and monolayer; **c** preparation of an anchored monolayer by PVD of CNBD onto Co_3_O_4_(111) at a sample temperature of 350 K, i.e. above the multilayer desorption temperature (orange squares: the total intensity, green circles: intensity of ν(OCO)_s_ band of anchored CNBD). All spectra were referenced to the clean sample

In the next step, we investigated the growth of CNBD films on Co_3_O_4_(111). In Fig. 2a, infrared reflection absorption spectra are displayed, which were recorded during the deposition of a multilayer film at 110 K. At the initial stage of deposition, the IR spectra are dominated by a broad band at 1400 cm^−1^, while the ν(C = O) band of the carboxylic acid at 1700 cm^−1^ is the most intense feature at larger exposure. The band at 1400 cm^−1^ is attributed to the symmetric stretching mode ν_s_(OCO) of a surface-bound carboxylate^24,29,30^. It indicates that CNBD anchors to the surface by deprotonation and formation of a chelating surface carboxylate that is attached to the surface Co^2+^ ions. After completion of the anchored monolayer, a multilayer of non-anchored CNBD starts to grow, as indicated by the appearance of the ν(C = O) band of the CNBD (see inset in Fig. 2a).

The thermal behavior of the CNBD multilayer film was probed by temperature-programmed (TP) IRAS, as shown in Fig. 2b. Here, IR spectra were recorded continuously, while the film was heated at a constant rate of 2 K^.^min^−1^. We observe a sudden decrease of all bands of the free CNBD at 280 K, which we attribute to desorption of the multilayer. The characteristic ν_s_(OCO) band of the surface-bound carboxylate is visible up to 570 K, indicating that the anchored CNBD monolayer resides on the surface up to this temperature. At higher temperature, several weaker bands are observed, which we attribute to decomposition products of CNBD formed at higher temperature. The behavior is consistent with other carboxylate films, studied previously on the same surface^24,31^.

As the anchored CNBD monolayer is stable between 280 K and 570 K, it should be possible to prepare a pure anchored monolayer film by PVD at surface temperatures in this range. The corresponding experiment is shown in Fig. 2c, where CNBD was deposited at a sample temperature of 350 K. All bands show a saturation behavior, with the ν_s_(OCO) band of the surface carboxylate being the dominating feature. We conclude that an anchored monolayer is formed. No indication is observed for the adsorption of CNBD molecules that are not anchored to the surface.

### Photochemical conversion of CNBD

**Fig. 3:**
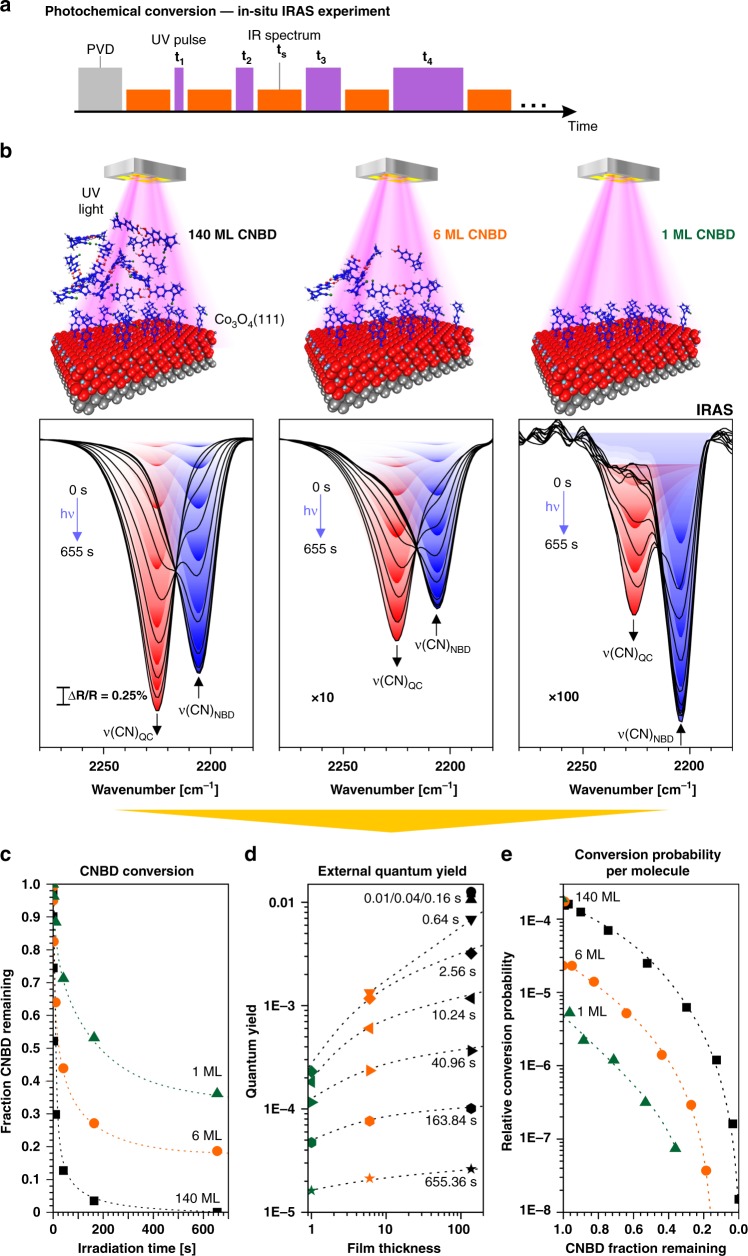
Photochemical conversion of CNBD films on an ordered Co_3_O_4_(111) surface: **a** the experimental procedure used in the photochemical IRAS experiment; **b** spectral region of the CN stretching bands of CNBD and CQC during UV irradiation for a thick multilayer film (140 ML), a thin multilayer film (6 ML), and an anchored monolayer film (1 ML). All spectra were referenced to the clean sample; **c** the fraction of residual CNBD as a function of irradiation time (green triangles: 1 ML, orange circles: 6 ML, black squares: 140 ML); **d** external quantum efficiency as a function of film thickness and irradiation time (circles: 0.01 s, squares: 0.04 s, triangles (top): 0.16 s, triangles (bottom): 0.64 s, diamonds: 2.56 s, triangles (left): 10.24 s, triangles (right): 40.96 s, hexagons: 163.84 s, stars: 655.36 s); **e** photoconversion probability per molecule as a function of conversion and film thickness (green triangles: 1 ML, orange circles: 6 ML, black squares: 140 ML s)

In the next step, we investigated the photoconversion of CNBD films using a photochemical UHV IRAS setup that was recently developed by some of the authors (see Fig. 1b). The experimental procedure is illustrated in Fig. 3a. The film was exposed to exponentially increasing doses of UV light (irradiation times: 0.01 s, 0.04 s, 0.16 s,…, 655.36 s), with each step followed by the acquisition of an IR spectrum. We used a UV source with a wavelength of 365 nm with an estimated power density of 920 mW^.^cm^−2^ at the sample surface^32^. The development in the spectral region of the ν(CN) band is shown in Fig. 3b for a thick multilayer film (140 monolayer equivalents, ML), a thin multilayer film (6 ML), and a single-anchored monolayer (1 ML). In all cases, we observe the disappearance of the ν_CNBD_(CN) band at 2204 cm^−1^ and the appearance of the ν_CQC_(CN) band at 2225 cm^−1^ upon irradiation, clearly showing that conversion of CNBD to CQC is possible, both in the multilayer and in the monolayer regimes.

Next, the photochemical conversion was analyzed quantitatively. We show three quantities that were calculated from the above spectra: the fraction of CNBD remaining as a function of irradiation time (Fig. 3c), the external quantum yield, i.e., the number of converted CNBD molecules per incident photon (Fig. 3d), and the photoconversion probability per molecule (Fig. 3e; See Supplementary Discussion for details).

The data show that the photoconversion reaction in the CNBD films strongly depends on two experimental parameters, the film thickness and the fraction of converted CNBD. The photoconversion is the fastest for thick CNBD films and for low conversion levels.

In order to unravel the origin of these dependencies, we consider the conversion in the thick multilayer in more detail. The reaction probability per molecule (see Fig. 3e) decreases rapidly with increasing conversion. Close to full conversion, it finally becomes very low and the uncertainty becomes large because of the low concentration of residual CNBD. One effect that contributes to this decrease is the different orientation of the CNBD molecules with respect to the electric field of the incident UV light. A quantitative analysis of this orientation effect (in which we assume random orientation in the multilayer) shows, however, that this effect only leads to a coverage dependence, which is much weaker than one observed experimentally (see Supplementary Discussion for details). Therefore, we suggest that the coverage dependence is mainly caused by support effects, i.e., by the underlying Co_3_O_4_(111) substrate. Support effects can reduce the conversion probability, for example by quenching of excited CNBD through energy transport to the support or by generation of hot electrons and electron–hole pairs, which trigger the backconversion of CQC to CNBD. Previously, we have observed a similar substrate effect in condensed films of non-functionalized NBD^32^.

Interestingly, the initial photoconversion probability also decreases with decreasing film thickness (see Fig. 3e). Experimentally, we observe a decrease by a factor of 40 ( ± 50%) between the 140 ML and the 1 ML films. This effect may be caused by the preferential molecular orientation in the monolayer, intermolecular interactions which modify the absorption spectrum, and screening of the electric field of the incident UV light at the metallic Ir substrate. A quantitative analysis of this electric-field effect shows that variations of the conversion rate by up to a factor of 30 are possible throughout a sufficiently thick film (see Supplementary Discussion for details). Therefore, it is likely that the electric-field effect largely contributes to the variation of the initial photoconversion probability, along with the support effects mentioned above. The importance of the latter is illustrated by the strong decrease of the photoconversion probability for the monolayer film, with increasing conversion (see Fig. 3e). Here, the conversion probability decreases by 2 orders of magnitude between 0% and 60% conversion. As molecular orientation effects typically play a lesser role in the anchored monolayer (where the molecules commonly adopt a similar orientation, in contrast to the randomly oriented multilayer), we assume that the decrease in the transition probability is mainly caused by support effects (i.e., by quenching or backconversion to CNBD).

### Thermally activated backconversion

**Fig. 4:**
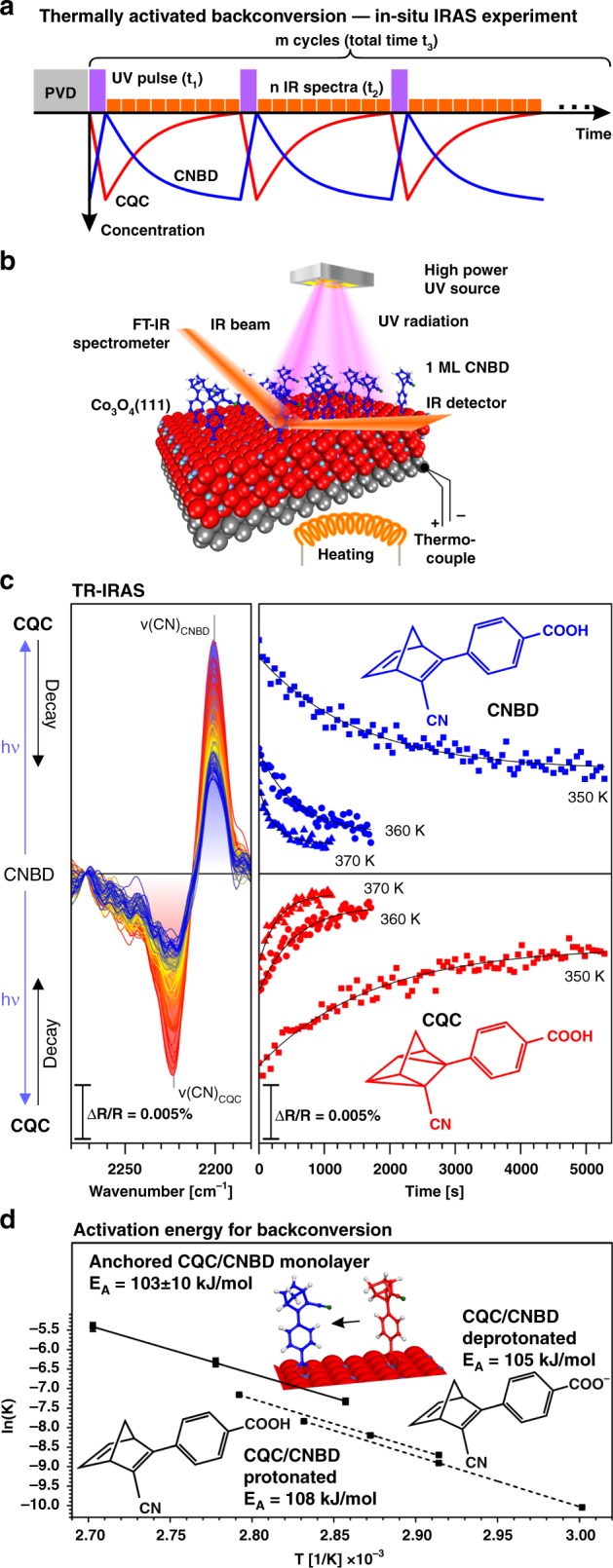
Thermally activated backconversion of a CNBD monolayer on Co_3_O_4_(111): **a** the experimental procedure and (**b**) experimental setup used in the in-situ IRAS experiment; **c** spectra and intensities of the CN stretching bands of CNBD and CQC during photochemical conversion and thermally activated backconversion, referenced to the anchored CNBD monolayer (squares: 350 K, circles: 360 K, triangles: 370 K); **d** Arrhenius plot comparing the kinetic parameters for backconversion of the anchored CNBD monolayer and free CNBD in solution (protonated and deprotonated form) (anchored: connected by straight line, in solution: connected by dashed line)

In the next step, we investigated the thermally activated backconversion of the anchored CQC monolayer. The experimental procedure is illustrated in Fig. 4a, b. After preparation of the CNBD monolayer, the film was exposed to a UV pulse (60 s), after which the thermally activated backconversion was recorded by time-resolved IRAS.

As the monolayer IR bands are extremely weak (ΔR/R ~0.01%), the photoconversion-decay cycles were repeated for 15 h and the corresponding IR data were accumulated. Such isothermal photoconversion-decay measurements were performed at temperatures between 350 and 370 K.

The experimental data are shown in Fig. 4c (right panel) in form of IR difference spectra (see the Methods section for details). The decrease of the negative ν_CQC_(CN) band at 2225 cm^−1^ and the increase of the positive ν_CNBD_(CN) band at 2204 cm^−1^ as a function of time clearly proves that anchored CQC is thermally back-converted to CNBD. With increasing sample temperature, we observe that the rate of backconversion becomes faster (Fig. 4c, right panel). The data allow us to derive the activation energy for thermally activated backconversion in the anchored CQC monolayer (see Fig. 4c), yielding a value of 103 ± 10 kJ^.^mol^−1^. In order to investigate whether the anchoring reaction has an effect on the backconversion, we also measured the kinetics of the backconversion for CQC in solution (toluene), both in the protonated form (CQC, carboxylic acid) and in the deprotonated form (CQC, carboxylate). The activation barriers (CQC protonated: 108 kJ^.^mol^−1^, CQC deprotonated: 105 kJ^.^mol^−1^, see Supplementary Methods for details) are very close to the value found for the anchored monolayer. We conclude that, within the accuracy of our experiments, the anchoring reaction has no effect on the activation barrier for backconversion.

### Stability of the photoswitchable monolayer

**Fig. 5:**
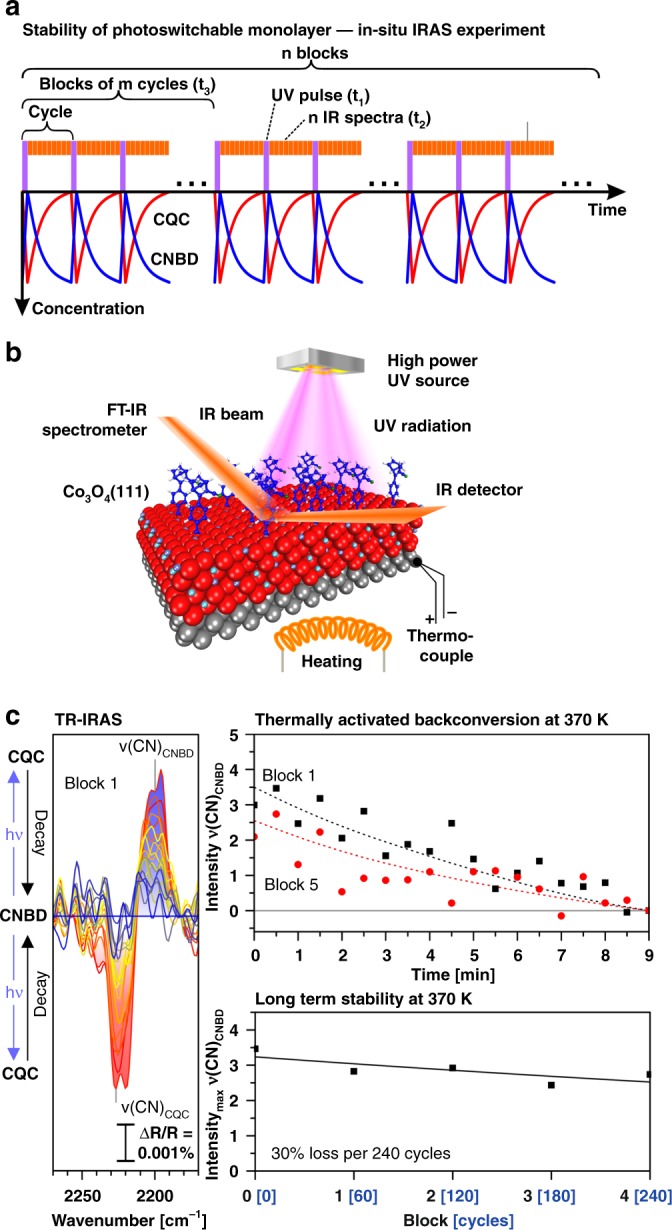
Stability of the CNBD monolayer on Co_3_O_4_(111) during (back-)conversion: **a** the experimental procedure of repeated photochemical conversion and thermally activated backconversion and (**b**) experimental setup used in the in-situ IRAS experiment; **c** IR spectra in the CN stretching frequency region of CNBD and CQC during photochemical conversion and thermally activated backconversion and development as a function of time, referenced to the anchored CNBD monolayer (black squares: block 1, red circles: block 5)

Finally, we tested the stability of the anchored CNBD/CQC monolayer during repeated energy storage and release cycles. The experimental procedure is illustrated in Fig. 5a, b. The anchored CNBD monolayer was exposed to pulses of UV light (60 s), followed by a decay period during which the backconversion was monitored by time-resolved IRAS (30 s per IR spectrum, 18 spectra). The photoconversion-decay sequence was repeated for a total duration of 50 h (300 cycles, 6000 IR spectra). The data (averaged in blocks of 60 cycles to obtain better signal/noise ratio) are shown in Fig. 5c. Over the total duration of the experiment, we observed a decay of the band intensities of CQC and CNBD by ~30%. Assuming that the degradation follows an exponential behavior, this value corresponds to a loss of 0.15% per storage and release cycle. Comparing the peak height of the ν_CNBD_(CN) band in Fig. 5c to the monolayer spectrum (see Fig. 2c), we estimate that ~10% of the NBD monolayer is converted per UV pulse. This corresponds to a loss of 1.5% per converted NBD molecule, i.e., to a reversibility of 98.5% per energy storage and release cycle in the anchored film.

In conclusion, we assembled a solar-energy-storing organic-oxide hybrid interface by anchoring a tailor-made photoswitch to an atomically defined oxide surface. The synthesized norbornadiene derivative 2-cyano-3-(4-carboxyphenyl)norbornadiene (CNBD) was linked to an atomically defined Co_3_O_4_(111) surface by PVD under UHV conditions. We monitored the photoconversion of CNBD to the quadricyclane derivative CQC and the thermally activated backconversion in situ using a photochemical surface IR spectroscopy experiment. We determined the quantum efficiencies and showed that photoconversion is possible even in a single-anchored CNBD monolayer on Co_3_O_4_(111). The anchored CNBD monolayer can be charged and discharged with high reversibility (98.5% reversibility), and the activation barrier for the thermally activated backconversion (103 ± 10 kJ^.^mol^−1^) is not affected by the anchoring reaction. The results show that it is possible to assemble a monolayer of NBD photoswitches on a semiconducting oxide interface without affecting the functionality of the photoswitch. The model interface presented in this work will enable fundamental studies on well-defined hybrid interfaces for solar-energy storage and conversion. An important next step toward a functional energy storage device would be to trigger the energy-release process electrochemically instead of thermally.

## Methods

### Experimental setup

See Supplementary Methods for details.

### Preparation of the Co_3_O_4_(111) film

The ordered cobalt oxide thin films were prepared on an Ir(100) single crystal (MaTeck, purity 99.999%) following an adapted method based on the procedure described by Heinz and Hammer^27^. The crystal was cleaned by cycles of Ar^+^ ion bombardment (Linde 6.0). Annealing at 1370 K for 3 min led to formation of the Ir(100)−(5 × 1) reconstructed surface which was checked by LEED. Successively, annealing in oxygen (5 × 10^−8^ mbar, Linde 5.0) at 1270 K for 3 min and cooling to 370 K in O_2_ yielded an Ir(100)−(2 × 1)O reconstructed surface as confirmed by LEED. Cobalt (Alfa Aesar, purity 99.95%, 2 mm diameter rod) was deposited for 20 min at a sample temperature of 265 K in O_2_ atmosphere (1 × 10^−6^ mbar) at a rate of 2 Å/min (Co metal equivalent) as determined by the QCM, yielding a Co_3_O_4_ film of ~8 nm thickness. An ordered Co_3_O_4_(111) film was formed in two annealing steps, at 520 K in oxygen atmosphere for 2 min and at 670 K in UHV for 10 min, as confirmed by LEED^25–27^.

### PVD of CNBD

CNBD was evaporated from a glass crucible loaded into a home built Knudsen cell. Prior to the first experiment, the system was separated from the main chamber by a gate valve, pumped by a separate high vacuum line, and baked for 24 h. Before deposition, the evaporator was preheated to 370 K before the gate valve was opened to start the deposition.

During deposition, IR spectra were acquired at a rate of 1 spectrum/min and a spectral resolution of 4 cm^−1^. The spectra were referenced to the background of the clean sample. For the temperature-programmed experiment, IR spectra were recorded at a rate of 1 spectrum/min while heating the sample at a rate of 2 K/min. Damping of the signal with increasing temperature was compensated for, following a procedure described by Xu et al.^31^, by normalization of the acquired spectra.

### Photochemical conversion in UHV

All photochemical experiments in UHV were performed with a home-built high-intensity UV source in the vicinity of the sample. In brief, we used a high-power LED (Seoul Viosys, CUN6AF4A, 2.35 W), which yields a photon flux density of 1.68 × 10^−18^ cm^−2^^.^s^−1^ at a wavelength of 365 nm, corresponding to a power density of 910 mW^.^cm^−2^. Further information can be found in the literature^32^. During all photoconversion experiments, the sample was cooled to 110 K. The UV source was operated by an external power supply (TDK Lambda Z + 200) triggered by the IR spectrometer (Bruker OPUS 7.2) or manually. For each sample, nine illumination steps were applied, such that the total illumination time was increasing exponentially (0.01 s, 0.04 s, 0.16 s, 0.64 s, 2.56 s, 10.24 s, 40.96 s, 163.84 s, 655.36 s). After each illumination step, an IR spectrum was recorded with an acquisition time of 10 min.

### Thermally activated backconversion

Backconversion in the anchored CNBD film was recorded at three different temperatures, i.e., 350 K, 360 K, 370 K. At each temperature, the sample was irradiated for 1 min followed by a decay period (89 min, 29 min, 19 min) during which IR spectra were recorded (2 spectra/min). The irradiation/decay cycles were repeated for 15 h at each temperature (10, 30, 45 cycles), and the data were accumulated over the cycles to improve the signal/noise ratio. Background spectra were taken before the measurement at each temperature step. The rate constants for backconversion were determined from exponential fits of the maximum peak heights between ν(CN)_CNBD_ and ν(CN)_CQC_.

### Stability test

Stability of the anchored CNBD film was tested by applying 300 cycles of illumination (1 min) and decay (9 min) at 370 K. IR spectra were recorded at a rate of 2 spectra/min (total acquisition time 50 h, 6000 spectra in total). To improve the signal/noise ratio, the IR spectra were averaged over blocks of 60 spectra (5 blocks). The data were analyzed for the peak height of the ν(CN)_CNBD_ band. The loss per cycle was estimated assuming an exponential decay.

### Transmission IR data

Transmission IR spectra of CNBD and CQC were recorded in FTIR-grade KBr (≥ 99%, Sigma Aldrich) using a FTIR spectrometer (Bruker VERTEX 80 v) at a spectral resolution of 2 cm^−1^ (acquisition time 1 min). For the spectrum of CQC, the CNBD sample was irradiated in an external cell by UV light (Seoul Viosys, CUN6AF4A, 365 nm, distance to sample 2 cm, illumination time 10 min).

### Synthesis

**Fig. 6:**
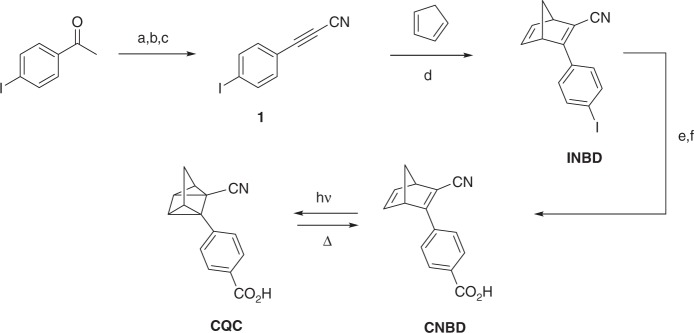
Synthetic sequence for CNBD-CQC photoswitch couple. Conditions: (**a**) POCl_3_, DMF, 3 h; (**b**) I_2_, NH_3(aq)_, CHCl_3_, 3 h; (**c**) NaOH_(aq)_, THF, 4 h; (**d**) chlorobenzene, BHT, 120 °C, 20 h; (**e**) *i*PrMgCl^.^LiCl, THF, −41 °C, 1 h; CO_2(g)_, −41 °C – RT, 16 h, followed by HCl_(aq)_

The molecular photoswitch CNBD was synthesized in a multistep sequence starting from commercially available 4-iodoacetophenone. This included an initial three-step transformation of the acetyl functionality to the corresponding propiolonitrile. Direct conjugation of the nitrile to the acetylene activated **1** toward a Diels–Alder [4 + 2π] cycloaddition reaction with cyclopentadiene to afford **INBD** in excellent yield. Knöchel conditions were finally used to promote an iodine–magnesium exchange and trapping of the carbanion with carbon dioxide generated CNBD (see Fig. 6).

All intermediates and products were characterized by ^1^H NMR and ^13^C NMR. The final products **CNBD** and **CQC** were characterized by UV–vis spectroscopy, including quantum yields for the photochemical conversion. Thermodynamic and kinetic data regarding the backconversion from CNBD to CQC were determined by differential scanning calorimetry (DSC) and kinetic UV–vis studies. The structure of CNBD was determined by single-crystal X-ray crystal structural analysis. All details of synthesis and characterization are given in the Supplementary Methods.

### DFT calculations

See Supplementary Methods for details.

## Supplementary information

Supplementary Information

Peer Review

## Data Availability

All experimental data are available from the corresponding author upon request.
