# Supplementary material for: Solar energy storage at an atomically defined organic-oxide hybrid interface
Source: Nat Commun. 2019 Jun 3;10:2384. doi: 10.1038/s41467-019-10263-4 (PMC6546758; doi:10.1038/s41467-019-10263-4)
Supplement: Supplementary file 1 — Supplementary Information [file 41467_2019_10263_MOESM1_ESM.pdf]

Supplementary Information

Solar energy storage at an atomically defined organic-oxide hybrid interface

Schuschke *et al.*

Supplementary Methods

Experimental setup. All IRAS experiments were performed in an UHV system (base pressure 1×10^{-10} mbar) consisting of a preparation chamber and a main chamber. The former contains an ion gun (Specs IQE 11/35), electron beam evaporators (Focus EFM3), a gas dosing system, a quartz crystal microbalance (QCM, Inficon SQM-160), a LEED optics (Specs ErLEED 150) and a quadrupole mass spectrometer (QMS, Blazers Quadstar 422). The main chamber is equipped with a programmable gas dosing system, various evaporation sources, a QMS (Hiden Analytical Hal 3F), and a home-built high-intensity UV source.[1] IR spectra were recorded with a vacuum Fourier-transform infrared (FTIR) spectrometer (Bruker VERTEX 80v) and a liquid nitrogen cooled mercury cadmium telluride (MCT) detector, connected to UHV chamber via differentially pumped KBr windows.

DFT calculations. DFT calculations were performed using the TURBOMOLE suite v7.2.[2] The exchange-correlation functional of Perdew, Burke and Ernzerhof[3] was used together with the def2-TZVP basis set of Weigend and Ahlrichs.[4] Dispersion interactions were modeled using the correction scheme D3 of Grimme[5] with a Becke-Johnson damping.[6] To accelerate the calculations RI-J[7] and MARI-J[8] approximations were applied. Frequency calculations were performed within the harmonic approximation. Data were visualized using the software Avogadro(V1.1.1)[9] and QVibeplot(V1.6.0).[10]

Synthesized compounds. *General:* Tetrahydrofuran was distilled from a sodium/benzophenone couple. All other chemicals were used as purchased from commercial sources. Purification of products was carried out by flash chromatography on silica gel (40−63 µm, 60 Å). Thin-layer chromatography (TLC) was carried out using aluminum sheets precoated with silica gel. Infrared (IR) spectra were recorded on a Perkin-Elmer Frontier FT-IR instrument as films evaporated from $CDCl_3$ onto an ATR attachment. All melting points and heat release of neat CNBD were recorded on a Mettler Toledo DSC 2 apparatus. ^{1}H NMR (400 MHz) and ^{13}C NMR (100 MHz) spectra were recorded on a Varian 400 MHz instrument using the residual solvent as the internal standard ($CDCl_3$, ^{1}H 7.26 ppm and ^{13}C 77.16 ppm). All chemical shifts are quoted on the δ scale (ppm) and all coupling constants (J) are expressed in Hz. All solution based spectroscopic

measurements were performed in a 1-cm path length cuvette scanning the wavelength from 700 to 290 nm on either a Cary 50 Bio or a Cary 100 UV-vis spectrophotometer, coupled with Peltier temperature control. Photoswitching for bulk conversion to CQC was performed using a Vilber Lourmet TLC lamp at 610 μw/cm^3 with a wavelength of 365 nm. Photoswitching at wavelength 310 nm was performed using Thorlabs M310L3 LED lamps. The thermal back reaction was performed by heating the sample (cuvette) by a Peltier unit in the UV-vis spectrophotometer. Quantum yields were measured by a published procedure in a high concentration regime (absorption above 2 at 300 nm) using potassium ferrioxalate and *tris*-phenanthroline iron (II) complex as a chemical actinometer.[11] The cuvette was irradiated perpendicularly in a fixed setup with stirring ensuring no movement during the experiment, using a Thorlabs LED lamp M300L4 with an attached collimator. HRMS spectra were acquired by atmospheric pressure chemical ionisation (APCI) using an Agilent 1260 Infinity instrument fitted with an Agilent 6120 quadrupole. Elemental analyses were performed at London Metropolitan University.

3-(4-Iodophenyl)propiolonitrile (1):

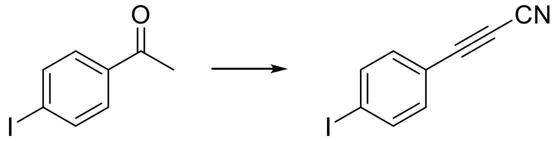

Supplementary Figure 1: Synthesis of compound **1**.

Alkyne **1** was made according to a previously reported protocol.[12] To stirring ice cooled DMF (100 mL, 1.29 mol) $POCl_3$ (15 mL, 160 mmol) was slowly added under a nitrogen atmosphere. The ice bath was removed and the vessel allowed to stir for 15 min. The vessel was re-immersed in ice, 4'-iodoacetophenone (12.51 g, 50.8 mmol) was added, and the flask was heated to 50 °C for 3 h. The cooled solution was poured into 20% aqueous NaOAc (300 mL) and then allowed to cool overnight. The solid was collected by suction filtration and washed with water (3 x 100 mL). Subsequently, the solid was dissolved in $CHCl_3$ (200 mL), and I_2 (12.00 g, 47.2 mmol) and 28% aqueous NH_3 (100 mL) were added to this stirring solution. After stirring for 3h at RT, saturated aqueous NaS_2O_3 (200 mL) was added and the phases were separated. The organic phase was dried over Na_2SO_4, filtered and the solvent was removed *in vacuo*. The residue was taken up in THF (200 mL), and aqueous NaOH (2.39 g, 59.8 mmol, in 10 mL H_2O) was added to this stirring solution. After 4 h, saturated $NaHCO_3$ (200 mL) was added, the mixture extracted with Et_2O (2 x 200 mL) and the combined organics washed with saturated brine (100 mL). The organic extracts were dried over Na_2SO_4, filtered and the solvent removed. The residue was purified by flash column chromatography (gradient elution of CH_2Cl_2/petroleum spirit 1:4 to CH_2Cl_2/petroleum spirit 3:7) to obtain **1** (8.40 g, 65%) as a light orange solid. R_f = 0.62 (CH_2Cl_2/petroleum spirit 3:7). M.p. = 139.7-140.1 °C. IR = 2273, 2254, 2204, 2143, 1642, 1580, 1572 cm^{-1}. ^{1}H NMR ($CDCl_3$, 400 MHz): δ = 7.78 (d, J = 8.7 Hz, 2H), 7.32 (d, J = 8.7 Hz, 2H) ppm. ^{13}C NMR ($CDCl_3$, 100 MHz): δ = 138.3, 134.6, 117.1, 105.5, 99.4, 82.1, 64.4 ppm. MS (GCMS, +ve): m/z = 253 ($M^{\bullet+}$). Analysis calculated for C_9H_4IN (253.04); C 42.72, H 1.59, N 5.54; found C 42.86, H 1.44, N 5.68.

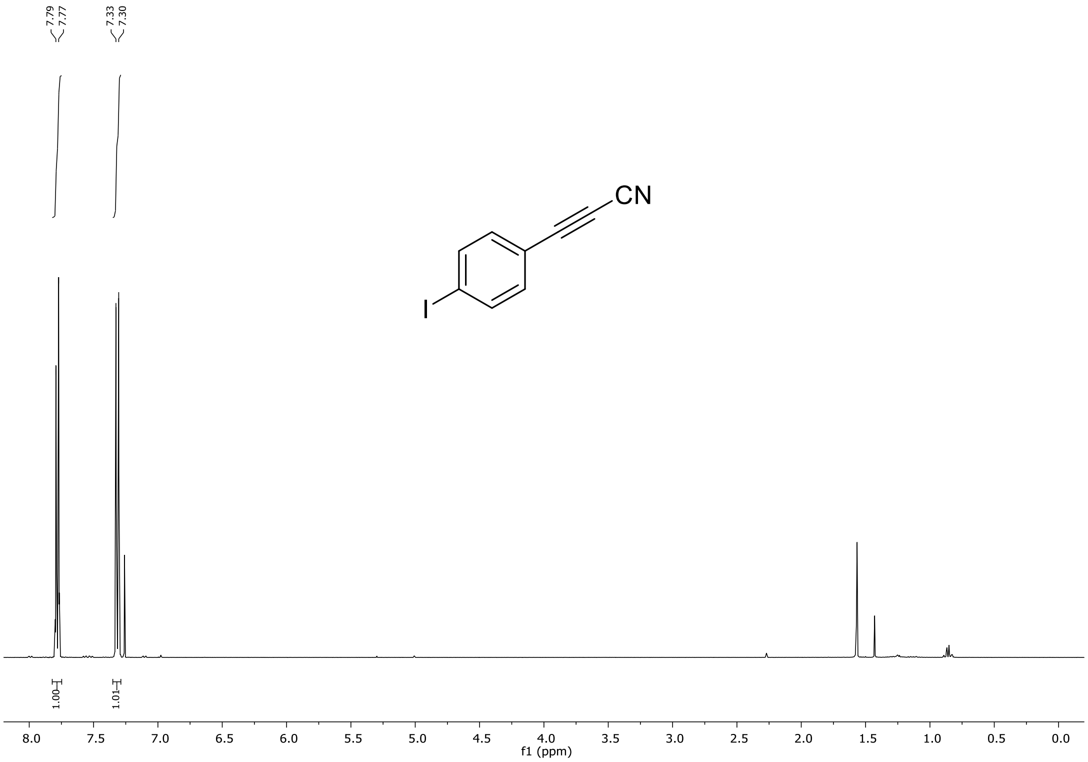

Supplementary Figure 2: ^{1}H NMR (400 MHz, $CDCl_3$) spectrum of **1**.

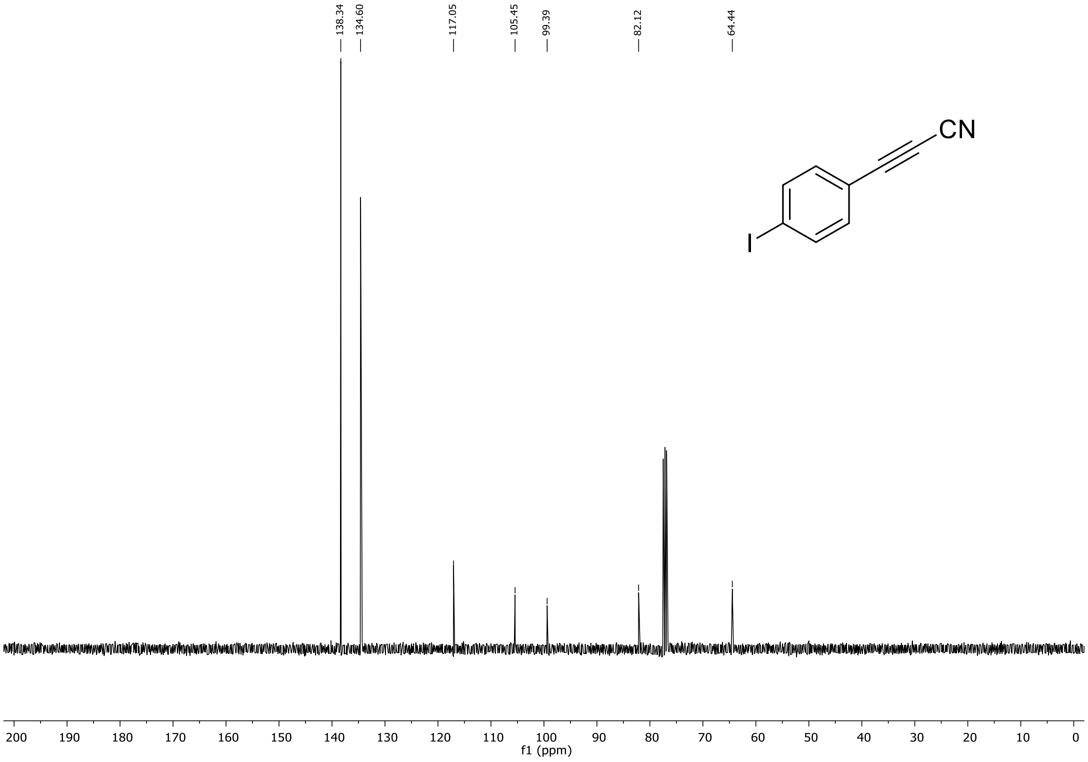

Supplementary Figure 3: ^{13}C NMR (100 MHz, $CDCl_3$) spectrum of **1**.

3-(4-Iodophenyl)norbornadiene-2-carbonitrile (INBD):

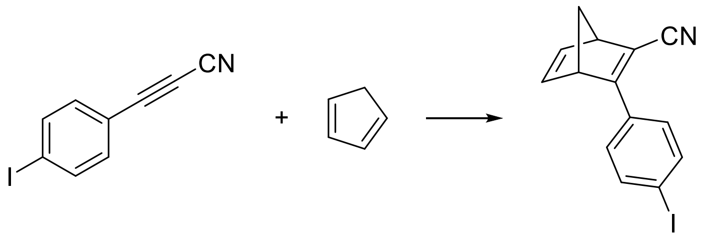

Supplementary Figure 4: Synthesis of **INBD**.

A vial suitable for microwave reactions was charged with cyclopentadiene (8 mL, 95 mmol), **1** (5.75 g, 22.7 mmol), BHT (20 mg) and chlorobenzene (7 mL). The vial was sealed and heated to 120 °C for 20 h. The resulting reaction mixture was directly subjected to flash column chromatography (CH_2Cl_2/petroleum spirit 2:3) to give **INBD** (6.68 g, 92%) as a pale yellow oil. R_f = 0.30 (CH_2Cl_2/*n*-heptane 2:3). IR = 3124, 3070, 2994, 2981, 2943, 2923sh, 2871, 2196, 1589sh, 1580, 1559 cm^{-1}. ^{1}H NMR ($CDCl_3$, 400 MHz): δ = 7.76 (d, J = 8.7 Hz, 2H), 7.44 (d, J = 8.7 Hz, 2H), 6.93 (ddd, J = 5.2, 3.0, 0.7 Hz, 1H), 6.85 (ddd, J = 5.2, 3.1, 0.8 Hz, 1H), 4.07 (ddtd, J = 3.1, 2.5, 1.6, 0.7 Hz, 1H), 3.94 (ddtd, J = 3.0, 2.5, 1.6, 0.8 Hz, 1H), 2.28 (dt, J = 6.9, 1.6 Hz, 1H), 2.20 (dt, J = 6.9, 1.6 Hz, 1H) ppm. ^{13}C NMR ($CDCl_3$, 100 MHz): δ = 169.9, 143.2, 140.3, 138.2, 132.6, 128.0, 118.2, 118.0, 96.5, 71.5, 55.2, 54.1 ppm. HRMS (APCI, +ve) calculated for $C_{14}H_{11}IN$ ($[M+H]^+$): 319.9931; exp 319.9932.

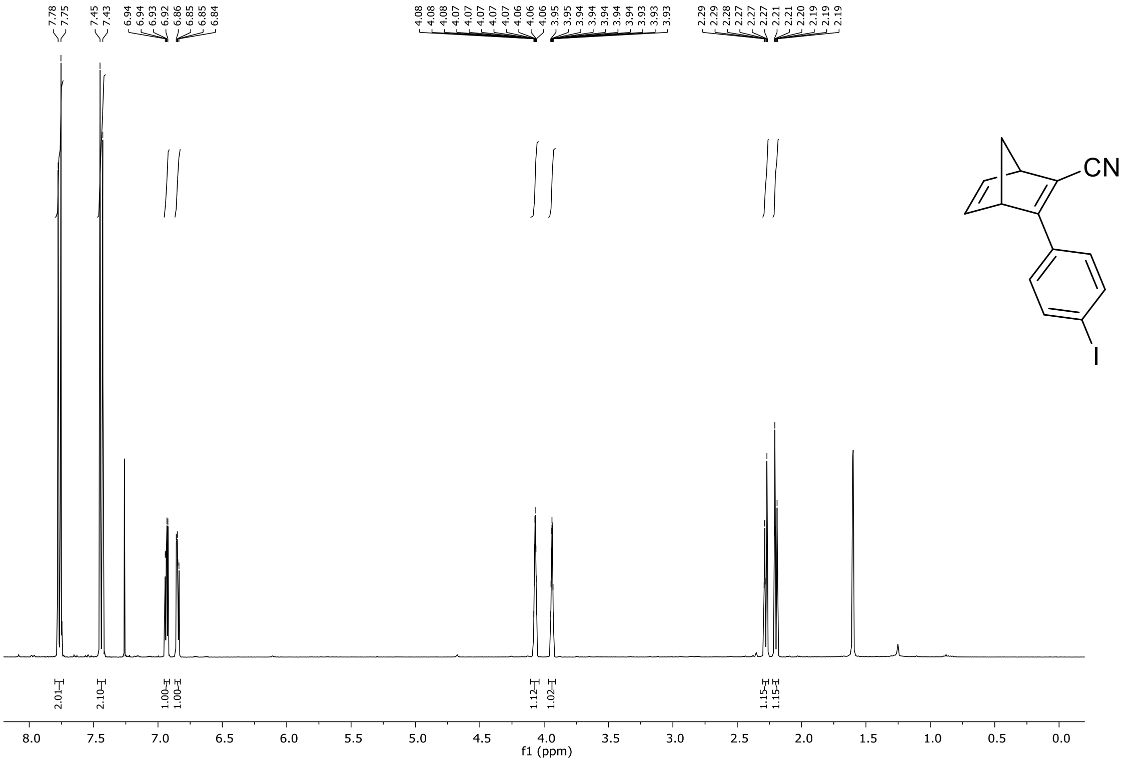

Supplementary Figure 5: ^{1}H NMR (400 MHz, $CDCl_3$) spectrum of **INBD**.

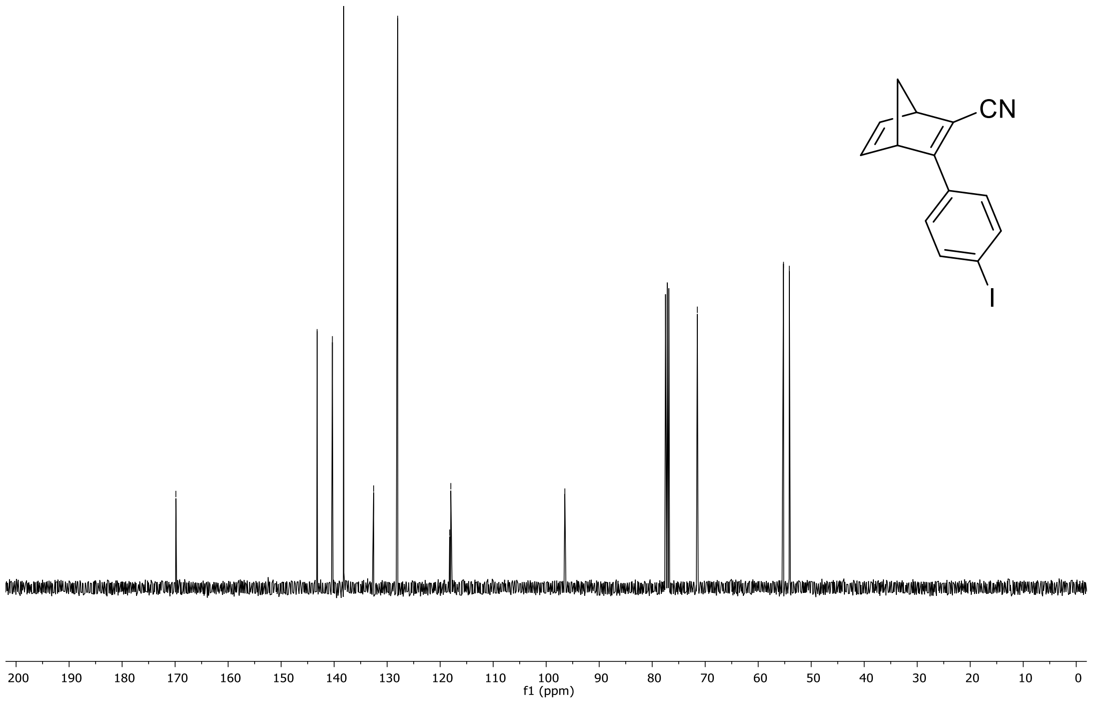

Supplementary Figure 6: ^{13}C NMR (100 MHz, $CDCl_3$) spectrum of **INBD**.

2-Cyano-3-(4-carboxyphenyl)norbornadiene (CNBD):

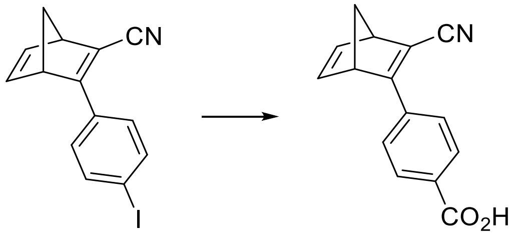

Supplementary Figure 7: Synthesis of **CNBD**.

To a stirring solution of **INBD** (6.67 g, 20.9 mmol) in dry distilled THF (300 mL) at -41 °C, , was added a solution of *iso*-propylmagnesium chloride. LiCl (18 mL, 1.3M in THF, 23.4 mmol) was added under an argon atmosphere and the resulting solution stirred for 1 h. CO_2 was bubbled through the solution using a balloon containing dry ice (ca. 10 g) and a needle immersed into the reaction solution. The cold bath was removed and the stirring was continued overnight. The reaction was quenched with addition of 1M aqueous HCl (200 mL) and the vessel diluted with Et_2O (200 mL). The phases were separated and the aqueous phase was extracted with Et_2O (200 mL). The combined ethereal extracts were combined, and H_2O (150 mL) and saturated aqueous Na_2CO_3 (50 mL) were added. The phases were separated and the aqueous phase was acidified with 1M HCl until pH = 1. This mixture was extracted with CH_2Cl_2 (3 x 200 mL) and the combined organics were dried over Na_2SO_4, filtered and the solvent removed *in vacuo*. The solid was crystallised (CH_2Cl_2/*n*-heptane) giving **CNBD** (3.59 g, 72%) as a crystalline white solid. M.p. = 191.5-192.8 °C. IR = 3071, 2996, 2984, 2945, 2872, 2830, 2670, 2550, 2197, 1685, 1607, 1584, 1562, 1558, 1509 cm^{-1}. ^{1}H NMR (400 MHz, $CDCl_3$): δ = 8.17 (d, J = 8.6 Hz, 2H), 7.81 (d, J = 8.6 Hz, 2H), 6.97 (ddd, J = 5.2, 3.0, 0.7 Hz, 1H), 6.91 (ddd, J = 5.2, 3.1, 0.8 Hz, 1H), 4.16 (ddtd, J = 3.1, 2.4, 1.6, 0.8 Hz, 1H), 3.99 (ddtd, J = 3.0, 2.4, 1.6, 0.7 Hz, 1H), 2.33 (dt, J = 7.0, 1.6 Hz, 1H), 2.25 (dt, J = 7.0, 1.6 Hz, 1H) ppm, CO_2H signal not visible presumably due to broadening. ^{13}C NMR (100 MHz, $CDCl_3$): δ = 171.3, 169.7, 143.2, 140.6, 138.0, 130.9, 130.3, 126.5, 120.5, 117.9, 71.7, 55.5, 54.4 ppm. HRMS (APCI +ve) calcd for $C_{15}H_{12}NO_2$ [$(M+H)^+$]: *m/z* = 238.0863; exp 238.0861. Analysis calculated (%) for $C_{15}H_{11}NO_2$ (237.26): C 75.94, H 4.67, N 5.90; found: C 76.19, H 4.71, N 5.68.

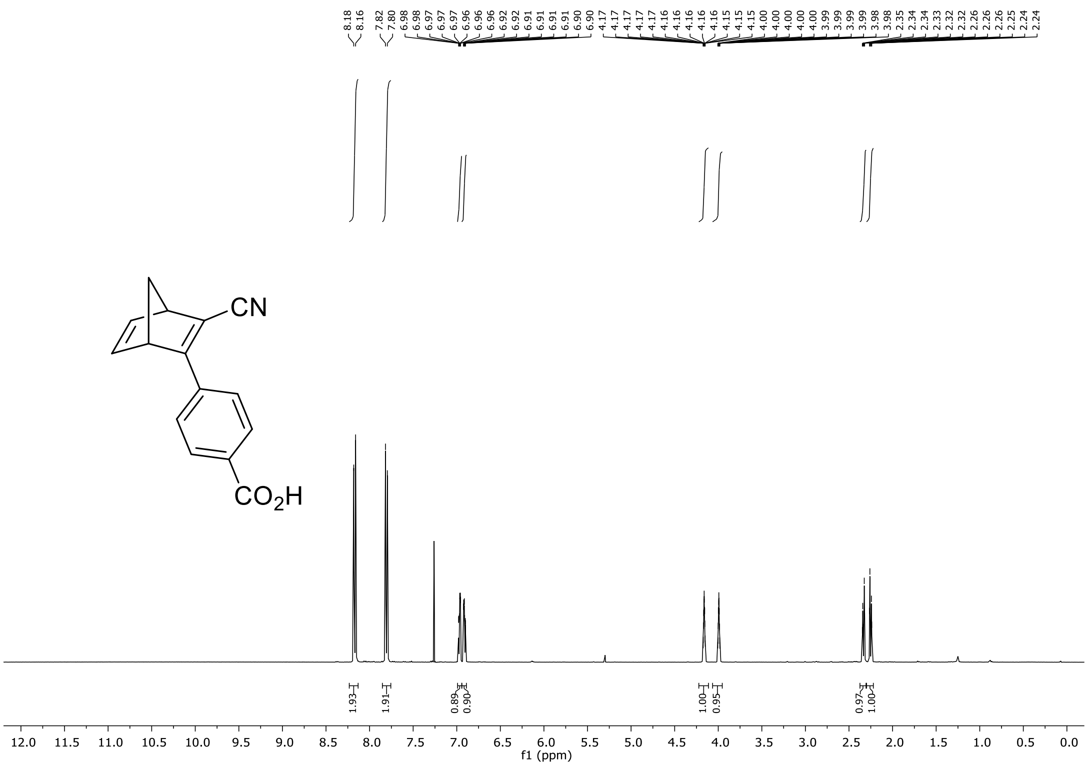

Supplementary Figure 8: ^{1}H NMR (400 MHz, $CDCl_3$) spectrum of **CNBD**.

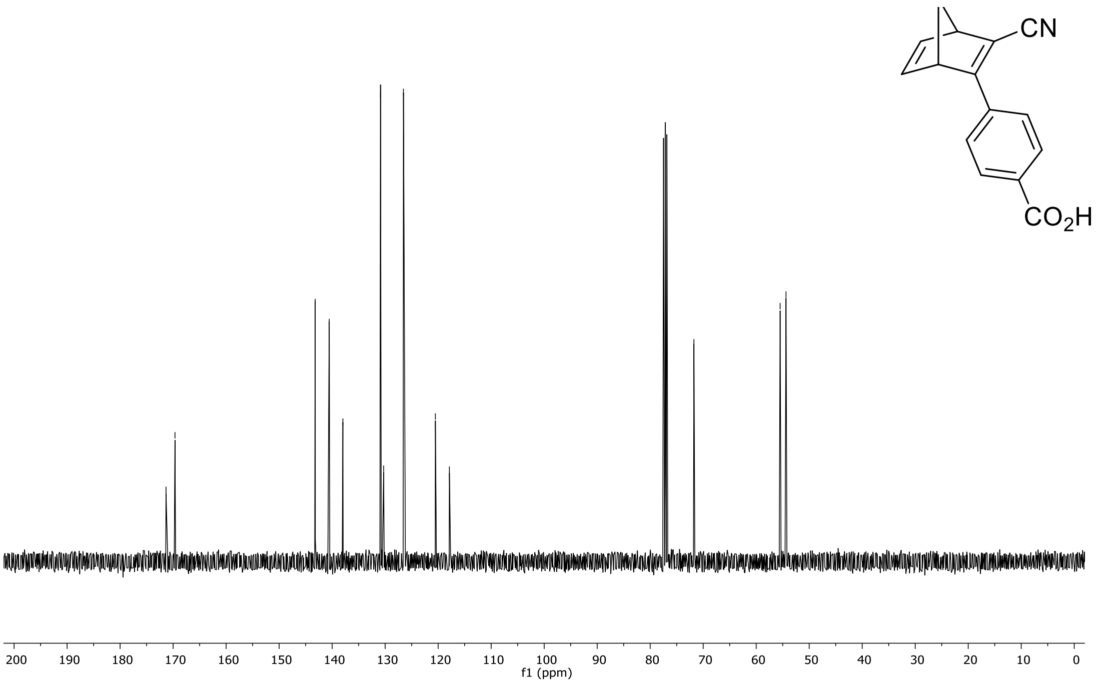

Supplementary Figure 9: ^{13}C NMR (100 MHz, $CDCl_3$) spectrum of **CNBD**.

2-Cyano-3-(4-carboxyphenyl)quadricyclane (CQC):

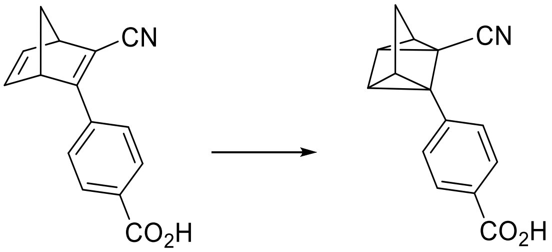

Supplementary Figure 10: Synthesis of CQC.

The corresponding quadricyclane (CQC) could be formed quantitatively by the irradiation of a $CDCl_3$ solution at wavelength 365 nm overnight. Concentration of the solution was performed cooled in an ice bath, firstly using a stream of nitrogen followed by high vacuum, The neat quadricyclane was used to conduct DSC measurements. IR = 3064, 2938, 2863, 2674, 2550, 2216, 1681, 1608, 1566, 1520 cm^{-1}. ^1H NMR (400 MHz, $CDCl_3$): δ = 11.99 (br s, 1H), 8.05 (d, J = 8.7 Hz, 2H), 7.28 (d, J = 8.7 Hz, 2H), 2.70 (dd, J = 4.9, 2.6 Hz, 1H), 2.49 (dt, J = 12.0, 1.4 Hz, 1H), 2.44 (dq, J = 4.9, 1.4 Hz, 1H), 2.41 (dd, J = 5.0, 2.6 Hz, 1H), 2.28 (dt, J = 12.0, 1.4 Hz, 1H), 2.03 (dq, J = 5.0, 1.4 Hz, 1H) ppm. ^{13}C NMR (100 MHz, $CDCl_3$): δ = 171.9, 143.2, 130.6, 127.2, 125.4, 119.3, 35.4, 33.7, 32.5, 31.3, 26.8, 24.1, 14.2 ppm.

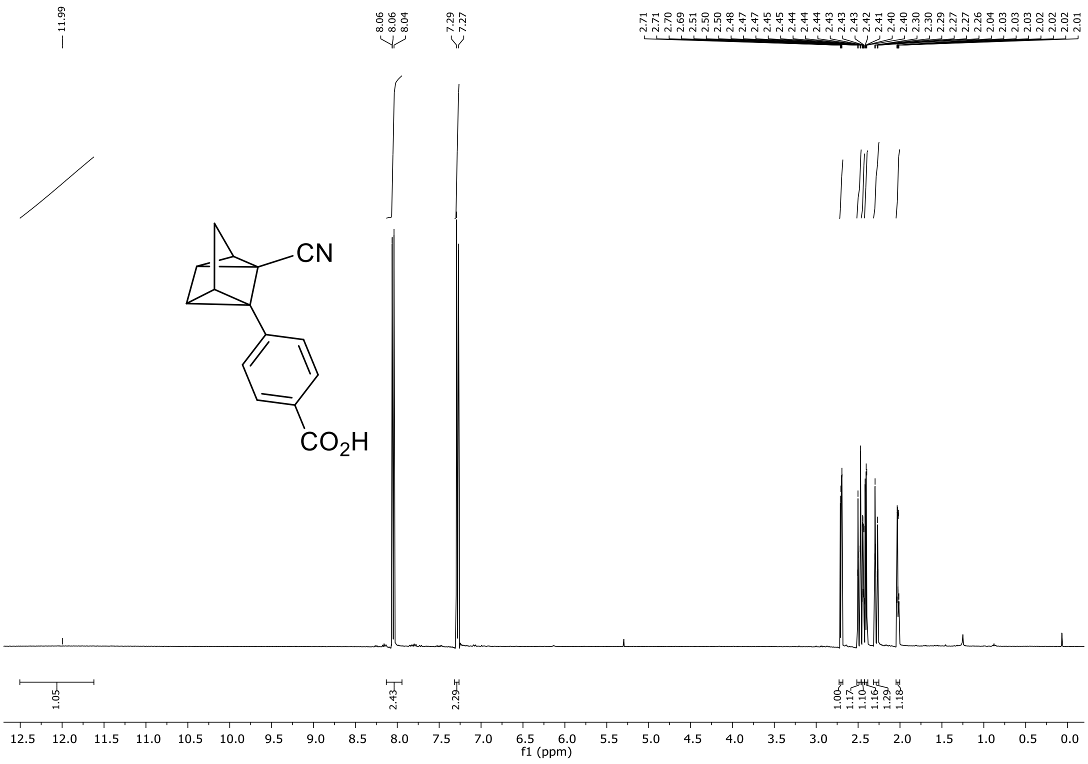

Supplementary Figure 11: ^{1}H NMR (400 MHz, $CDCl_3$) spectrum of **CQC**.

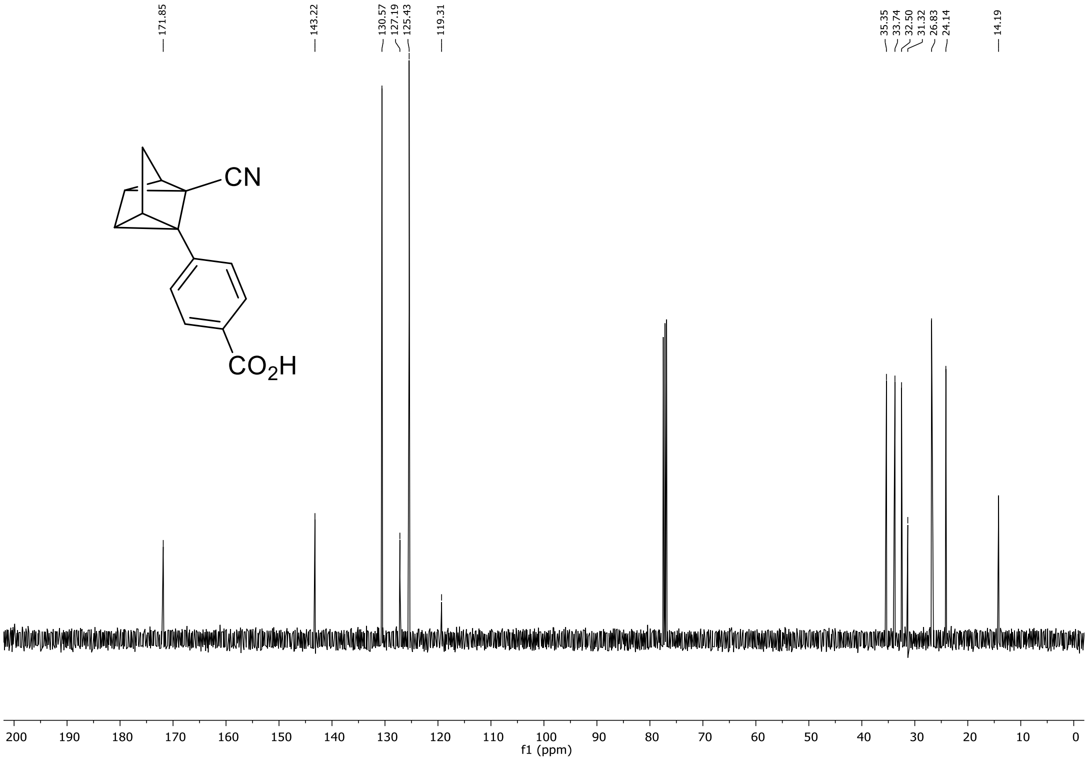

Supplementary Figure 12: ^{13}C NMR (100 MHz, $CDCl_3$) spectrum of **CQC**.

Spectroscopic analysis. *UV-vis studies of CNBD.* Extinction coefficient at 319 nm for CNBD calculated as the average of four toluene solutions (11497, 11090, 10993, 10617) gives 11049 $M^{-1}cm^{-1}$. All characterisation by UV-vis for CNBD was made in toluene solutions.

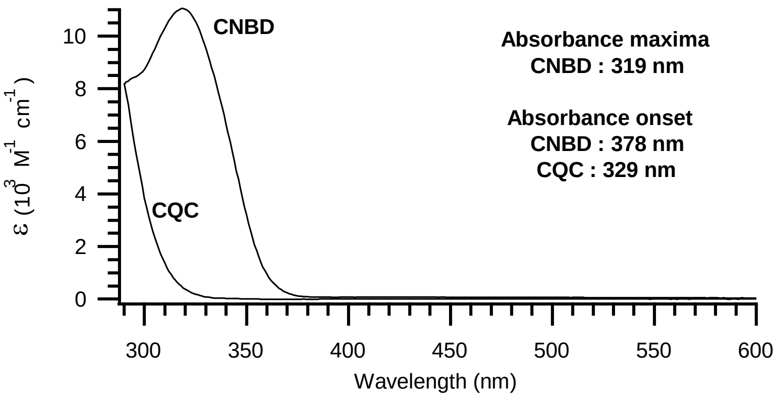

Supplementary Figure 13: Absorbance spectrum showing CNBD and CQC isomers.

Quantum yield for photoconversion of CNBD to CQC. The quantum yield for photoconversion of CNBD to CQC was calculated.

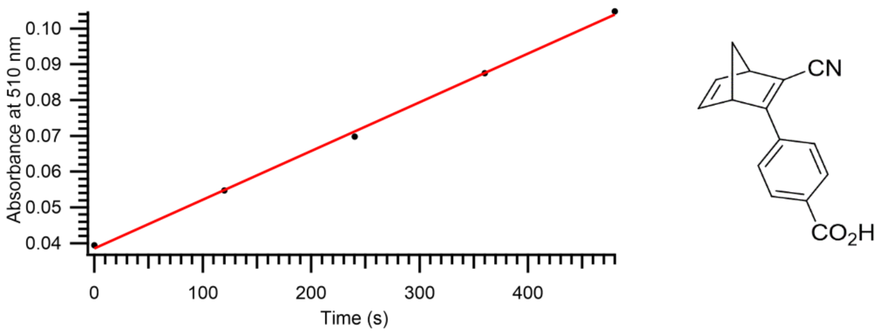

Supplementary Figure 14: Absorbance vs time, 300 nm LED, photon flux: 1.485 x 10^{-9} mol s^{-1}.

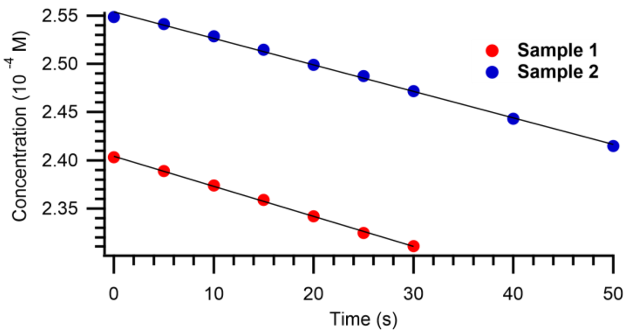

Supplementary Figure 15: Concentration vs. time, ϕ_1 = 56.66 %, ϕ_2 = 55.28 %, $\phi_{average}$ = 56 %. (red circles: sample 1, blue circles: sample 2)

Kinetics for reconversion of CNBD to CQC. Back-conversion in solution: 3 mL of CNBD solution (8×10^{-5} M) in toluene were illuminated to yield CQC. Subsequently, the reconversion was monitored at 333, 343, and 353 K by UV-vis. In order to test the effect of deprotonation, 5µl trimethylamine was added and the back-conversion was investigated at 343, 348, and 358 K. The activation energy of the reconversion of CNBD in toluene was calculated in similar fashion to the UHV data, monitoring the absorbance maximum at 319 nm by UV-vis.

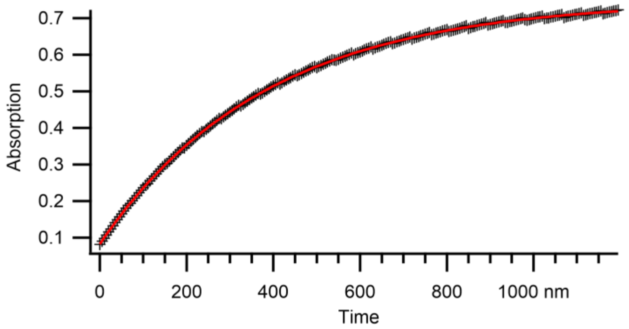

Supplementary Figure 16: Formation of CNBD at 60 °C.

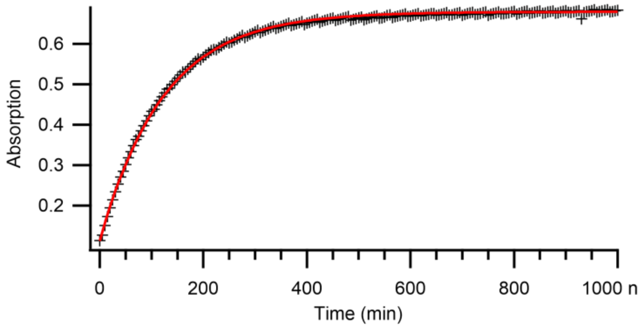

Supplementary Figure 17: Formation of CNBD at 70 °C.

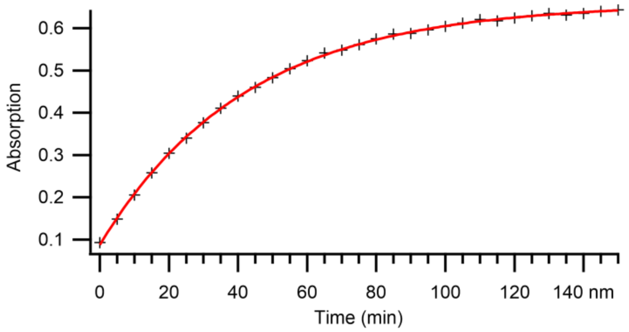

Supplementary Figure 18: Formation of CNBD at 80 °C.

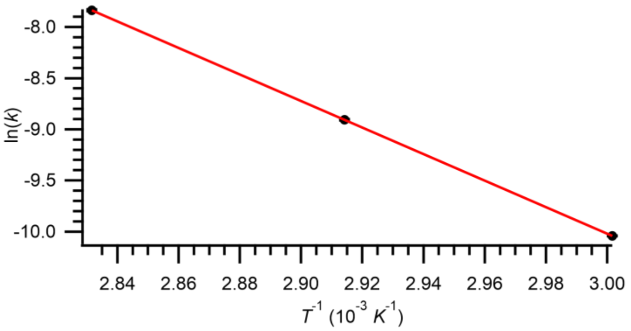

Supplementary Figure 19: Arrhenius plot yielding the values: A = 3.69 · 10^{12} s^{-1}, E_a = 107972 J mol^{-1}, $t_{½}$ = 18 days in toluene.

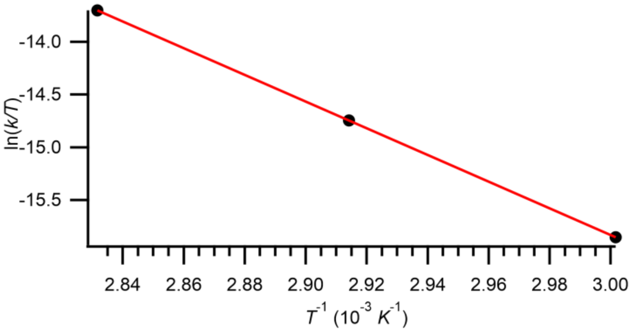

Supplementary Figure 20: Eyring plot giving the values: ΔH = 105.1 kJ/mol ΔS = -13.8 J mol^{-1} K^{-1}.

Differential Scanning Calorimetry (DSC) of CQC.

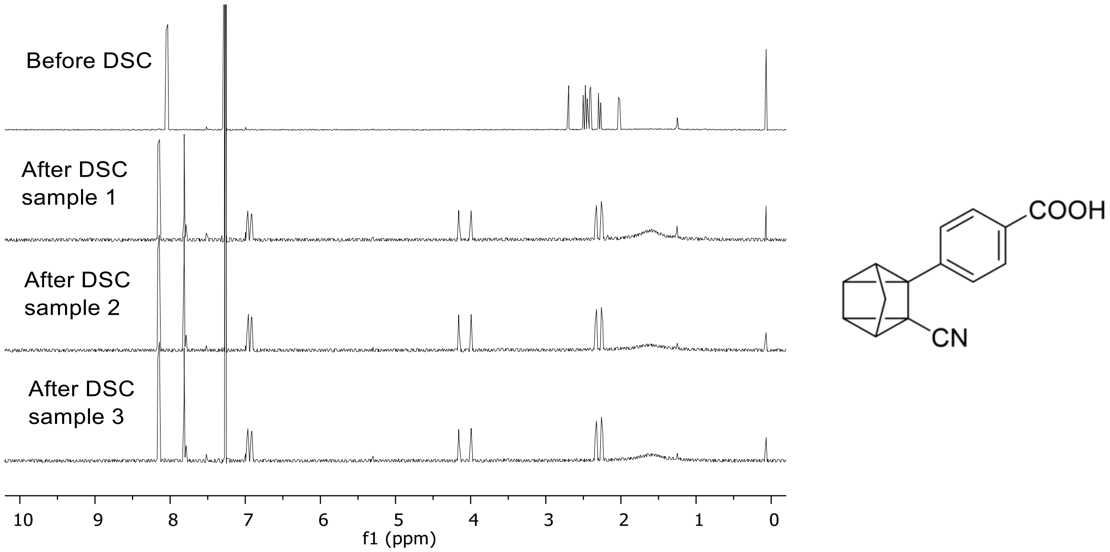

Supplementary Figure 21: H NMR (400 MHz, $CDCl_3$) spectra of CQC, before and after DSC

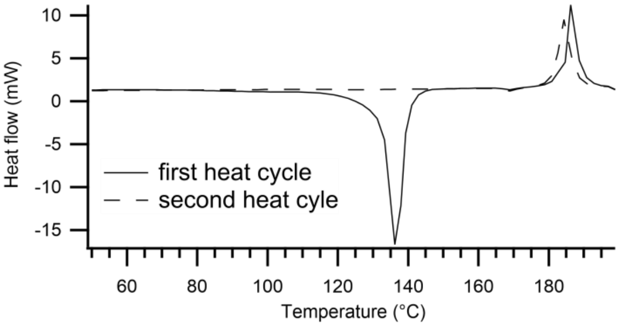

Supplementary Figure 22: DSC, heating rate 20 °C/min, amount 1.08 mg, baseline set at 87.69 – 159.28 °C, integration area: 87.69 – 159.28 °C, heat release: 362.70 kJ/kg

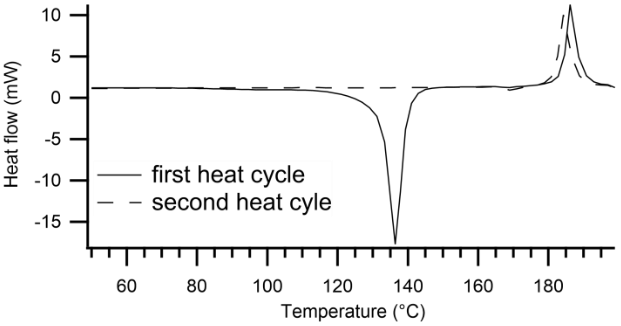

Supplementary Figure 23: DSC, heating rate 20 °C/min, amount 1.12 mg, baseline set at 87.04 – 184.18 °C, integration area: 87.04 – 184.18 °C, heat release: 364.78 kJ/kg

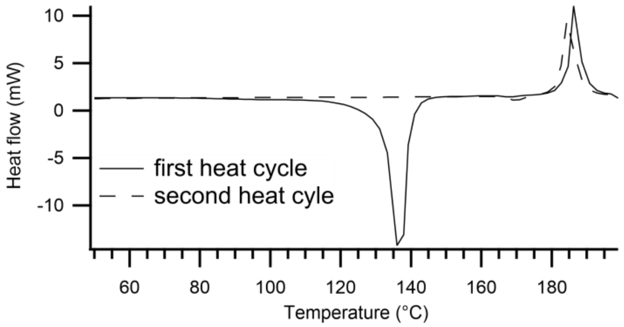

Supplementary Figure 24: DSC, heating rate 20 °C/min, amount 1.06 mg, baseline set at 81.27 – 151.53 °C, integration area: 81.27 – 151.53 °C, heat release: 360.84 kJ/kg

The calculations give an average heat release of 363 kJ/kg.

Single crystal X-ray crystal structure. A single crystal was mounted in paratone-N oil on a plastic loop. X-ray diffraction data were collected at 150(2) K on an Oxford X-calibur single crystal diffractometer using Mo Kα radiation.[13] The data set was corrected for absorption using a multi-scan method, and structure solved by direct methods using SHELXS-2014 and refined by full-matrix least squares on F2 by SHELXL-2014,[14, 15] interfaced through the program X-Seed.[16] All non-hydrogen atoms were refined anisotropically and hydrogen atoms were included as invariants at geometrically estimated positions. The X-ray experimental data and refinement parameters for CNBD are given below.

Crystal data for CNBD (cif labelled NBDacid)**.** $C_{15}H_{11}NO_2$, F.w. 237.25, monoclinic, $P2_1/c$, *a* 8.0554(3), *b* 17.4701(8), *c* 8.4495(4) Å, β 102.406(4)°, *V* 1161.32(9) Å^3, *Z* = 4, D_{calc} = 1.357 Mg/m^3, μ 0.091 mm^{-1}, *F*(000) 496, crystal size 0.62 × 0.22 × 0.17 mm^3, θ range for data collection 3.38 to 29.22°, Ind. reflns 2883, Obs. reflns 2260, R_{int} 0.0366, *GoF* 1.116, R_1 [I>2σ(I)] 0.0718, wR_2 (all data) 0.1613, largest diff. peak and hole 0.661 and -0.291 e.Å^{-3}.

Full details of the structure determination has been deposited with the Cambridge Crystallographic Data Centre as CCDC 1885135. Copies of this information may be obtained free of charge from The Director, CCDC, 12 Union Street, Cambridge CB2 1EZ, U.K. (fax, +44-1223-336-033; e-mail, deposit@ccdc.cam.ac.uk).

Perspective views of the single X-ray crystal structure of CNBD are shown below highlighting the NBD framework and the planarity of the chromophore. Crystals were grown from CH_2Cl_2/*n*-heptane and data collected as described above.

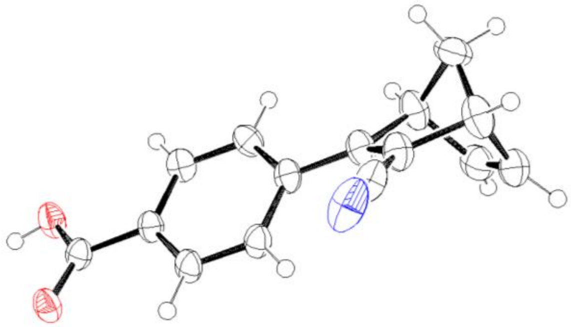

Supplementary Figure 25: Single crystal X-ray crystal structure of CNBD, perspective 1.

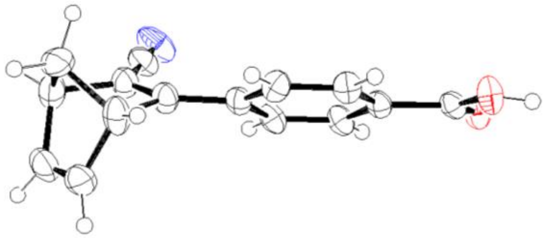

Supplementary Figure 26: Single crystal X-ray crystal structure of CNBD, perspective 2.

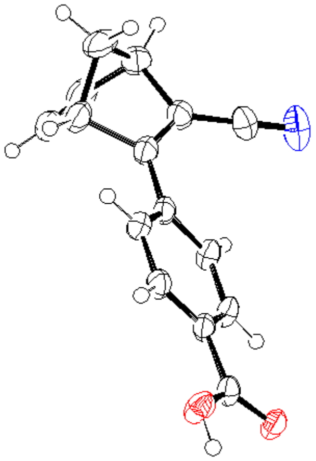

Supplementary Figure 27: Single crystal X-ray crystal structure of CNBD, perspective 3.

Supplementary Discussion

Calculation of quantum efficiency and conversion probability. The ν(CN) region of the IRAS data were fitted using the KolXPD software package. The density of CNBD molecules in one monolayer was assumed to be identical to the number of binding sites at the surface Co^{2+} ions, which is 3.6 nm^{-2}.[17] Other thicknesses were estimated from the deposition time relative to the monolayer value. The external quantum efficiency *QE* was calculated as

$$QE = \frac{n(\mathrm{CNBD_{consumed}})}{n_{\mathrm{photons}}} \quad (1)$$

where $n(\mathrm{CNBD_{consumed}})$ is the density of CNBD converted, and n_{photons} is the number of incident UV photons per area as calculated from the photon flux density and the irradiation time. The relative conversion probability per molecule *P* was calculated as

$$P = \frac{\Delta n(\mathrm{CNBD})}{\Delta n_{\mathrm{photons}} \times n(\mathrm{CNBD})} \quad (2)$$

where $\Delta n(\mathrm{CNBD})$ are the CNBD molecules converted per irradiation step and $\Delta n_{\mathrm{photons}}$ are the number of incident photons per irradiation step.

Effect of the molecular orientation on the conversion probability. We assume that the CNBD molecules are randomly oriented in the multilayer and so are the transition dipole moments μ over the sphere as illustrated below. The incident light is directed along the z axis and the not polarized.

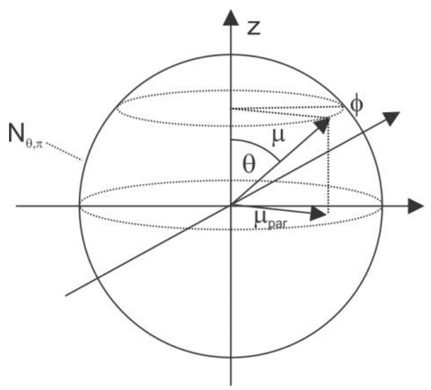

Supplementary Figure 28: Orientation of transition dipole moments within volume integral.

The fraction of transition dipoles $x(\vartheta..\pi/2)$ which have a polar angle between $\theta$and ${}^{\pi}/_{2}$ is:

$$x = \frac{1}{2\pi}\int_{\vartheta}^{\frac{\pi}{2}}\int_{0}^{2\pi} sin\vartheta \cdot d\theta d\varphi = cos\theta \tag{3}$$

The active component μ_{par} of the transition dipole moment $\mu(\theta)$ which is parallel to the xy plane is calculated as

$$\mu_{\mathrm{par}} = \mu \cdot \ sin\theta \tag{4}$$

The corresponding absorption probability (or photoconversion probability) is

$$P = c \cdot {\mu_{par}}^2 = c \cdot \mu^2 \ sin^2\theta \tag{5}$$

where c is an experimental constant.

Using the above result, the photoconversion probability can be expressed as a fraction of the total number of absorbers as:

$$\begin{aligned} P = c \cdot \mu^2 \ sin^2\theta &= c \cdot \mu^2 \ sin^2(arccosx) \\ &= c \cdot \mu^2(1 - cos^2(arccosx)) \\ &= c \cdot \mu^2(1 - x^2) \end{aligned} \tag{6}$$

Consequently, we would expect the transition probability to decrease to 75% of its original value at a conversion of $x = 0.5$ and to 19% of its original value at a conversion of $x = 0.9$. The experimental values are much larger, showing that the molecular orientation is not the dominating factor for coverage dependence of the photoconversion probability. Also the inhomogeneity of the light intensity over the irradiated area is by far too small to account for the very large variations in photoconversion probability, which exceed two orders of magnitude between $x = 0$ and $x = 0.9$.

Effective electric field at the CNBD/Co_3O_4(111) interface. The CNBD/Co_3O_4 interface is irradiated by UV light at normal incidence. Due to the low thickness of the Co_3O_4 film (8 nm), we consider the light reflection at the underlying Ir single crystal. From the Fresnel formula, the reflection coefficient at normal incidence is

$$r = \frac{E_R}{E} = \frac{n_1 - n_2 + ik_2}{n_1 + n_2 - ik_2} \quad (7)$$

where n_1 is the refractive index of the external medium, i.e. vacuum or CNBD film, and n_2 and k_2 are the real and the complex (absorption) part of the refractive index of the Ir crystal. From the above formula, the phase shift φ between the incident and the reflected wave is calculated as

$$\tan\varphi = \frac{2k_2 n_1}{{n_1}^2 - {n_2}^2 - {k_2}^2} \quad (8)$$

and the amplitude of the reflected wave is calculated as

$$|E_R| = |E|\sqrt{\frac{(n_1 - n_2 + ik_2)^2}{(n_1 + n_2 - ik_2)^2}} \quad (9)$$

Using the refractive index of Ir at 360 nm ($n_2 = 1.9$ and $k_2 = 3.3$) we calculate a phase shift of 154° at the vacuum interface ($n_1 = 1$), an amplitude of the reflected wave of $|E_R| = 0.78|E|$ and a resulting field at the interface of $|E_R + E|_{\mathrm{interface}} = 0.30|E|$.

For an organic film with $n_1 = 1.5$, we calculate $|E_R| = 0.70|E|$, so that the maximum field strength above the surface will be $|E_R + E|_{\max} = 1.70|E|$. As the UV absorption will be proportional to E^2, the maximum ratio between absorption in the thick film and monolayer film will be

$$\left(\frac{|E_R + E|_{\max}}{|E_R + E|_{\mathrm{interface}}}\right)^2 = \left(\frac{1.70}{0.30}\right)^2 \approx 32 \quad (10)$$

The ratio compares well with the ratio between the initial reaction probabilities in the thick multilayer film and in the monolayer.

Supplementary Tables

Supplementary Tables 1: IR Spectra of CNBD and CQC.

**Wavenumber [cm^{-1}] CNBD**			**Wavenumber [cm^{-1}] CQC**			**Assignment**
**Transmission**	**IRAS**	**DFT**	**Transmission**	**IRAS**	**DFT**	
3500	3200	2689		3200	2689	ν(OH)
2980	3000	3077+3073				$\nu(CH)_{NBD}$
			3054	3081	3102	$\nu(CH)_{ph}$
2945	2957	3060	2927	2937	3029	$\nu(CH_2)_{as}$
2869	2874	2997	2868	2865	2982	$\nu(CH_2)_{sym}$
2665+2551	2685+2563		2663+2543	2683+2562		$\nu(OH)_{CN}$
2202	2204	2209	2216	2225	2239	ν(CN)
1686	1726+1695	1661	1686	1722+1691	1661	ν(C=O)
1613	1609	1606				$\nu(C{=}C)_{ph}$
1592+1563	1563	1546	1607+1564 +1516	1612+1569 +1519	1606+1557	$\nu(C{=}C)_{ph}+\delta(CH)_{ph}$
1506	1509	1496				$\delta(CH)_{ph}$
1423	1425	1449	1423	1429	1447	δ(OH)
1407	1412	1408	1412	1415	1408	$\nu(C{=}C)_{ph}+\delta(CH)_{ph}$
			1380	1380	1387	ν(CC)
		1346	1322	1326	1343	$\nu(C{=}C)_{ph}+\delta(OH)$
1316+1291	1324+1297	1326+1307	1295	1298	1316	$\nu(CC)+\delta(CH)_{ph}+\delta(OH)$
			1271	1272	1289+1284	$\delta(CH)_{ph}+\delta(CH)_{QC}$
1228	1229		1230	1228		
1184	1184	1179	1182	1184	1172	$\delta(CH)_{ph}$
			1156	1157	1148	$\delta(CH)_{QC}$
1127	1108		1127	1107		
1031	1027	1039	1041	1031	1039	γ(OH)
940	942		970	969		
860	861	851				$\gamma(CH)_{ph}$
			852+827	852+829	855+840	$\gamma(CH)_{QC}+\gamma(CH)_{ph}$
766	780	786				$\gamma(CH)_{NBD}$
			761	770	762	$\gamma(CH)_{ph}$
730	727	719				$\gamma(CH)_{NBD}$

Supplementary Table 1: List of IR vibrations.

Supplementary References

1. Schwarz M., *et al.* A Simple High-Intensity UV-Photon Source for Photochemical Studies in UHV: Application to the Photoconversion of Norbornadiene to Quadricyclane. *Rev. Sci. Instrum.* **90**, 024105 (2019).

2. University of Karlsruhe, Forschungszentrum Karlsruhe GmbH. Turbomole. Available from: http://www.turbomole.com (2013)

3. Perdew J.P., Burke K., Ernzerhof M. Generalized Gradient Approximation Made Simple. *Phys. Rev. Lett.* **77**, 3865-3868 (1996).

4. Weigend F., Ahlrichs R. Balanced basis sets of split valence, triple zeta valence and quadruple zeta valence quality for H to Rn: Design and assessment of accuracy. *Phys. Chem. Chem. Phys.* **7**, 3297-3305 (2005).

5. Grimme S., Antony J., Ehrlich S., Krieg H. A consistent and accurate ab initio parametrization of density functional dispersion correction (DFT-D) for the 94 elements H-Pu. *J. Chem. Phys.* **132**, 154104 (2010).

6. Grimme S., Ehrlich S., Goerigk L. Effect of the Damping Function in Dispersion Corrected Density Functional Theory. *J. Comput. Chem.* 2011, **32**, 1456-1465 (2011).

7. Weigend F. Accurate Coulomb-Fitting Basis Sets for H to Rn *Phys. Chem. Chem. Phys.* **8**, 1057-1065 (2006).

8. Sierka M., Hogekamp A., Ahlrichs R. Fast evaluation of the coulomb potential for electron densities using multipole accelerated resolution of identity approximation. *J. Chem. Phys.* **118**, 9136-9148 (2003).

9. Hanwell M.D., *et al.* Avogadro: an advanced semantic chemical editor, visualization, and analysis platform. *J. Cheminformatics* **4**, 17 (2012).

10. Laurin M. QVibeplot: A Program To Visualize Molecular Vibrations in Two Dimensions. *J. Chem. Educ.* **90**, 944-946 (2013).

11. Stranius K., Börjesson K. Determining the Photoisomerization Quantum Yield of Photoswitchable Molecules in Solution and in the Solid State. *Sci. Rep.* **7**, 41145 (2017).

12. Sharma P.K., Ram S., Chandak N. Transition Metal-Free Approach to Propynenitriles and 3-Chloropropenenitriles. *Adv. Synth. Catal.* **358**, 894-899 (2016).

13. Rigaku Oxford Diffraction. CrysAlisPro Software System, Version 1.171. 38.43d. Oxford, UK; 2015.

14. Sheldrick G. A short history of SHELX. *Acta Crystallogr. A* **64**, 112-122 (2008).

15. Sheldrick G. Crystal structure refinement with SHELXL. *Acta Crystallogr. C* **71**, 3-8 (2015).

16. Barbour L.J. X-Seed — A Software Tool for Supramolecular Crystallography. *Journal of Supramol.Chem.* **1**, 189-191 (2001).

17. Heinz K., Hammer L. Epitaxial cobalt oxide films on Ir(100)—the importance of crystallographic analyses. *J. Phys. Condens. Matter* **25**, 173001 (2013).
