# Supplementary material for: Solar energy storage at an atomically defined organic-oxide hybrid interface
Source: Nat Commun. 2019 Jun 3;10:2384. doi: 10.1038/s41467-019-10263-4 (PMC6546758; doi:10.1038/s41467-019-10263-4)
Supplement: Supplementary file 2 — Peer Review [file 41467_2019_10263_MOESM2_ESM.pdf]

Reviewers' comments:

Reviewer #1 (Remarks to the Author):

In a previous paper (ref. 22), (some of) the authors have introduced an extremely intriguing concept: to fabricate something like a DSSC, but with a photo-switchable molecule (NBD -> QC). This would have the advantage that no electrons would be injected into the semiconductor under open circuit conditions, making feasible a solar cell that can store energy.

As pointed out in the intro part, the key aspect would be the anchoring of the switchable molecule on the semiconductor electrode, ideally a true-and-tried oxide such as TiO2 anatase. This paper deals with this critical junction, proving the key requirement that the molecule remains switchable while anchored, and that the system is stable.

The work is technically flawless – the data are of very high quality, and the manuscript is well-written. (Addition of the cif file is just one nice touch.)

Particularly noteworthy is the approach of using not just some random oxide, but a well-defined model system under the most stringent (UHV) conditions. By exploiting an IRAS setup that yields elegant spectra, and synthesising an NDB derivative with special features (carboxylate anchoring group, photo-sensitizer and CN-moiety that shows a distinct red-shift upon isomerization), the authors could build up an intriguing model system.

There are other nice aspects as well: the interpretation of the – rather complex – vibrational spectra is aided by DFT calculations, and the characterization of the molecules, and evaluation of quantum efficiency is executed in an equally thorough manner as the UHV study.

The only weak point is that the oxide model system (Co3O4/Ir(100)) is not one that is easily usable by others: while the Libuda group has perfected its use, and it is well documented in the literature how to produce the oxide thin film, the preparation is involved and not as straightforward as, say a TiO2 single crystal that much more readily available and closer to the support material that will eventually be used. Also, as correctly pointed out in the manuscript, the observed drastic (by two orders of magnitude!) decrease in efficiency of photoswitching in the monolayer is possibly caused by polarization of the underlying metal substrate, and could be lessened by a dielectric material.

Nevertheless, the work is interesting and of high quality and can (except for the minor points mentioned below) essentially be published as-is.

Minor points:

- (optional) It would be good to state in each figure caption which reference spectra were taken for the IRAS measurements

- It would be good to mention the electronic structure of the Co3O4 thin film somewhere up front in the manuscript. Is it metallic? Semiconducting? What band gap?

- p. 8 states: "we consider the conversion in the thick multilayer in more detail. The reaction probability per molecule (see Figure 3e) decreases rapidly with increasing conversion and, finally, becomes immeasurably low." I don't understand this? It seems from Fig. 3c that virtually all molecules are converted after irradiation for 600 s? Isn't this the reason that the probability cannot be evaluated properly?

- While the system is very well-characterized spectroscopically, the statement p.10 "That all molecules adsorb in the same way", is in fact, not verified experimentally and should be weakened.

-- Ulrike Diebold, TU Wien

Reviewer #2 (Remarks to the Author):

The manuscript on solar energy storage at an atomically defined organic-oxide hybrid interface presents original work on the anchoring of a photoswitchable norbornadiene derivative onto an epitaxial Co3O4(111) film grown on Ir(100)under ultra high vacuum conditions. While there is a vast amount of literature on dye sensitized solar cells based on anchoring a dye onto an oxidic surface to convert absorbed light directly into a photo current the idea to convert solar energy into chemically stored energy via strucural changes of an immobilised organic molecule is novel. The manuscript is written in a stringent way and understandable also for a broader audience.

The main results presented are based on FT-IRRAS data supported by DFT calculations. The data are thoroughly presented. However, there are some minor questions: In Fig. 2 IR data are given for a multilayer of CNBD and a monolayer. The scales are the same (Delta R/R = 0,25%), but the intensity of the monolayer in Fig. 2a is much smaller than in Fig. 2c. The scale in Fig. 3 is even given in Delta R/R=0,5%, but the intensities are even much larger.

In order to discuss photoconversion efficiencies the authors analyse the IR data. The IR intensities only scale linearly for multilayers (Fig. 2a), but exhibit a linear behaviour only in a limited coverage regime (Fig. 2c)for submonolayers. The authors should comment on how they have taken this into account.

The authors discuss support effects when comparing monolayer with multilayer results and attribute the changes in efficiencies mainly to the underlying oxide. They should comment about the excitation wavelength in comparison to the band gap of the oxide and quantify their discussion. The excitation is above the band gap of the Co3O4 oxide so that an electron transfer is indeed (as they argue) possible from the oxide to the dye. Why did they choose this oxide and not an oxide with a larger band gap? Also the packing of the organic molecules may be important as coupling processes between the organic molecules may influence the data. Did the authors try to get LEED data from monolayers of the organic molecules to check about possible ordering (in contrast to the multilayer)? Is there anything known about shifts of the UV-spectra which may result in shifts of the spectrum compared with the unordered layer which have to be taken into account for calculating efficiencies? Single crystal UV/Vis data for the dye may be of help insted of solvent data.

Is there a reason why the excitation has been done in the low tailing regime (highly sensitive to possible shifts)and not closer to the maximum of the irradiation source as apparent when comparing the UV/Vis data of the dyes to the maximum of the LED used?

Can the authors give an outlook how to build a device in the future to harvest the chemical energy and to restore the device without using energy to heat?

All in all these are minor questions which the authors should be able to answer. Therefore I suggest to publish the manuscript after minor revision.

Manuscript NCOMMS-19-03156-T

Response of the Authors to the Referee Report and List of Revisions
First, we want to thank the referees for their positive feedback on the manuscript and their valuable remarks. After carefully considering the different points, we would like to address the specific comments, taking them in the order in which they appear in the reports.
"Referee comments are reproduced in italics"
Replies are given in standard font.
Changes made in the manuscript are highlighted in bold.

Referee 1:

In a previous paper (ref. 22), (some of) the authors have introduced an extremely intriguing concept: to fabricate something like a DSSC, but with a photo-switchable molecule (NBD -> QC). This would have the advantage that no electrons would be injected into the semiconductor under open circuit conditions, making feasible a solar cell that can store energy.

As pointed out in the intro part, the key aspect would be the anchoring of the switchable molecule on the semiconductor electrode, ideally a true-and-tried oxide such as TiO2 anatase. This paper deals with this critical junction, proving the key requirement that the molecule remains switchable while anchored, and that the system is stable.

The work is technically flawless – the data are of very high quality, and the manuscript is well-written. (Addition of the cif file is just one nice touch.)

Particularly noteworthy is the approach of using not just some random oxide, but a well-defined model system under the most stringent (UHV) conditions. By exploiting an IRAS setup that yields elegant spectra, and synthesising an NDB derivative with special features (carboxylate anchoring group, photo-sensitizer and CN-moiety that shows a distinct red-shift upon isomerization), the authors could build up an intriguing model system.

There are other nice aspects as well: the interpretation of the – rather complex – vibrational spectra is aided by DFT calculations, and the characterization of the molecules, and evaluation of quantum efficiency is executed in an equally thorough manner as the UHV study.

The only weak point is that the oxide model system (Co3O4/Ir(100)) is not one that is easily usable by others: while the Libuda group has perfected its use, and it is well documented in the literature how to produce the oxide thin film, the preparation is involved and not as straightforward as, say a TiO2 single crystal that much more readily available and closer to the support material that will eventually be used. Also, as correctly pointed out in the manuscript, the observed drastic (by two orders of magnitude!) decrease in efficiency of photoswitching in the monolayer is possibly caused by polarization of the underlying metal substrate, and could be lessened by a dielectric material.

Nevertheless, the work is interesting and of high quality and can (except for the minor points mentioned below) essentially be published as-is.

We appreciate the positive appraisal of the referee. The aim of the present study was to build a well-defined hybrid interface and demonstrate that the photoswitch remains operational when anchored. We perfectly agree that changing the oxide support would be an important next step and TiO_2 would be a natural choice. However, bulk TiO_2 single crystals are poorly compatible with IR spectroscopy in reflection experiments (the signal intensity is more than one order of magnitude lower than for thin oxide films on metals) and all TiO_2 film on metals that are available today are relatively poorly defined. In any case, finding alternative well-defined oxide electrodes is a challenge that we will address in our future work.

Minor points:

- (optional) It would be good to state in each figure caption which reference spectra were taken for the IRAS measurements.

We fully agree to the referee's comment.

We adapted the captions of figure 1, 2, 3, 4, and 5 accordingly.

- It would be good to mention the electronic structure of the Co3O4 thin film somewhere up front in the manuscript. Is it metallic? Semiconducting? What band gap?

We agree to the referee's comment and modified the text to clarify this point.

We added "semiconducting" on page 3, line 87.
We added the following sentence on page 5, line 122:
"Note that bulk Co_3O_4 is a semiconducting oxide with a band gap of 1.6 eV,[28] while STM studies suggest that the band gap is larger for the thin films used in this work.[26]"

- p. 8 states: "we consider the conversion in the thick multilayer in more detail. The reaction probability per molecule (see Figure 3e) decreases rapidly with increasing conversion and, finally, becomes immeasurably low." I don't understand this? It seems from Fig. 3c that virtually all molecules are converted after irradiation for 600 s? Isn't this the reason that the probability cannot be evaluated properly?

The referee is correct, in the sense that conversion probability becomes more difficult to measure close to the point of full conversion (i.e. after 600 s). Still, it is important to understand that the conversion probability per molecule is decreasing with increasing conversion, i.e., we are not dealing with a simple exponential decrease of the CNBD concentration. We modified the text to clarify this point.

We modified text on page 8, line 195:
"The reaction probability per molecule (see Figure 3e) decreases rapidly with increasing conversion. Close to full conversion, it finally becomes very low and the uncertainty becomes large because of the low concentration of residual CNBD."

- While the system is very well-characterized spectroscopically, the statement p.10 "That all molecules adsorb in the same way", is in fact, not verified experimentally and should be weakened.

We agree to the comment and rephrased the statement.

We changed the text on page 10, line 227:
"As molecular orientation effects typically play a lesser role in the anchored monolayer (where the molecules commonly adopt a similar orientation, in contrast to the randomly oriented multilayer), we assume that the decrease in the transition probability is mainly caused by support effects (i.e., by quenching or back-conversion to CNBD)."

Referee 2:
The manuscript on solar energy storage at an atomically defined organic-oxide hybrid interface presents original work on the anchoring of a photoswitchable norbornadiene derivative onto an epitaxial Co3O4(111) film grown on Ir(100)under ultra high vacuum conditions. While there is a vast amount of literature on dye sensitized solar cells based on anchoring a dye onto an oxidic surface to convert absorbed light directly into a photo current the idea to convert solar energy into chemically stored energy via strucural changes of an immobilised organic molecule is novel. The manuscript is written in a stringent way and understandable also for a broader audience.

The main results presented are based on FT-IRRAS data supported by DFT calculations. The data are thoroughly presented. However, there are some minor questions: In Fig. 2 IR data are given for a multilayer of CNBD and a monolayer. The scales are the same (Delta R/R = 0,25%), but the intensity of the monolayer in Fig. 2a is much smaller than in Fig. 2c.

The referee has spotted an interesting feature of monolayer growth at different temperatures. Previous work on anchored carboxylic acid monolayers (e.g. *Chem. Eur. J.* **2016**, *22*, 5384 – 5396, and *Langmuir* **2017***, 33*, 4178−4188) at different temperatures showed that the molecules can adopt different orientation at different growth temperatures. Due to the Metal Surface Selection Rule (MSSR) the intensity of the

symmetric COO stretching mode at 1423 cm^{-1} can, therefore, vary quite substantially. The dynamic dipole moments of other modes are less well aligned (e.g. as combinations of CC and CH vibrations in the wavenumber range around 1200 cm^{-1}) and, therefore, show similar signal intensities in the monolayer at 110 K and 300 K**.**

The scale in Fig. 3 is even given in Delta R/R=0,5%, but the intensities are even much larger.

This is a mistake we thank the referee for pointing it out.

We corrected Figure 3b: We changed the label at the scale bar to Delta R/R=0.25%.

In order to discuss photoconversion efficiencies the authors analyse the IR data. The IR intensities only scale linearly for multilayers (Fig. 2a), but exhibit a linear behaviour only in a limited coverage regime (Fig. 2c) for submonolayers. The authors should comment on how they have taken this into account.

It is correct that the band intensities grow non-linearly with exposure during the preparation of the monolayer. As mentioned above, the effect is mainly due to reorientation of the molecular film with coverage. For the photoconversion experiment we used fully saturated monolayers only. We assume that the changes in molecular orientation are minor upon photoconversion, because the molecular density in the film does not change and the CNBD and CQC are similar in size.

The authors discuss support effects when comparing monolayer with multilayer results and attribute the changes in efficiencies mainly to the underlying oxide. They should comment about the excitation wavelength in comparison to the band gap of the oxide and quantify their discussion. The excitation is above the band gap of the Co_3O_4 oxide so that an electron transfer is indeed (as they argue) possible from the oxide to the dye. Why did they choose this oxide and not an oxide with a larger band gap?

In the present work, our aim was to build an atomically defined organic/oxide interface to test the functionality of the photoswitch (which could be achieved). We agree that in the next step it will be important to investigate the role of the electronic structure of the oxide support. The photon energy for conversion of the present photoswitch is 3.4 eV which is, however, above the band gap of the most common semiconducting oxides (including TiO_2). In order to decouple the excitation of the photoswitch and the support it would be necessary to modify both the support and the design of the photoswitch. We plan to address this challenge in future work.

Also the packing of the organic molecules may be important as coupling processes between the organic molecules may influence the data. Did the authors try to get LEED data from monolayers of the organic molecules to check about possible ordering (in contrast to the multilayer)?

The referee addresses an important point. So fat, we were not able to obtain meaningful LEED data from the present system. In any case, LEED may not be the method of choice because of the sensitivity of organic/oxide interfaces to electron bombardment. We are optimistic, however, that some information on the growth and self-assembly of the CNBD films may become available in the future from STM studies under UHV conditions.

Is there anything known about shifts of the UV-spectra which may result in shifts of the spectrum compared with the unordered layer which have to be taken into account for calculating efficiencies? Single crystal UV/Vis data for the dye may be of help insted of solvent data.

Unfortunately, we were not able to acquire UV spectra from CNBD single crystals. However, concentration and solvent effects on UV spectra are typically very small, in comparison to the large width of the UV bands. The only effects that could have a dramatic influence on the position of the UV bands are those which directly affect the electronic structure of the molecule. Such changes occur, for instance, when changing the structure of the molecule but not when changing the molecular environment.
The chemical processes that could have affected the UV absorption spectrum of the CNBD the strongest in the present study, is the anchoring reaction. The latter involves deprotonation of the carboxylic acid group and, therefore, a direct change of the electronic structure of part of the molecule. Therefore, we investigated the effect of deprotonation on the UV spectrum of CNBD and found that, even for this quite dramatic modification, the effect on the UV spectrum is minor.

Is there a reason why the excitation has been done in the low tailing regime (highly sensitive to possible shifts) and not closer to the maximum of the irradiation source as apparent when comparing the UV/Vis data of the dyes to the maximum of the LED used?

The choice of the wavelength was due to the commercial availability of the LED sources. It is possible to use LEDs with shorter wavelength, however, the photon flux of these sources is much lower (factor 10 to 100), so that the resulting overall conversion efficiency would be worse than in the present case.

Can the authors give an outlook how to build a device in the future to harvest the chemical energy and to restore the device without using energy to heat?

In order to release the stored energy without heating, the energy release process can be triggered either catalytically or electrochemically. The catalytically triggered energy release has recently been demonstrated in an operational device.(Wang *et al., Energy Environ. Sci*. **2019**, *12,* 187-193, ref. 13) The concept of electrochemically triggered energy release has been demonstrated and discussed in a second publication.(Brummel *et al., ChemSusChem* **2016***, 9*, 1424-1432, ref. 22) Both works are discussed in the introduction. The great challenge is the electrochemically triggered conversion to electrical energy, which involves a series of challenges (including the ones that have been discussed above). In future work, we will aim to assemble NBD/oxide interfaces, similar as the one in the present work, and trigger the energy release via the electrode potential instead of heating. This is ongoing work, however, and we hope to be able to present first results in the near future.

To clarify this point, we have added the following sentence to the conclusion section on page 14, line 293:
"An important next step towards a functional energy storage device would be to trigger the energy release process electrochemically instead of thermally."

All in all these are minor questions which the authors should be able to answer. Therefore I suggest to publish the manuscript after minor revision.

REVIEWERS' COMMENTS:

Reviewer #2 (Remarks to the Author):

Most of the questions have been answered to my satisfaction. I only disagree with respect to the statement that UV-Vis spectra are only different in the solid state from the solvent state because of chemical changes due to the anchoring. J-aggregates or H-aggregates exhibit subtantially shifted spectra because of Davidov-splitting and modified selection rules because of interactions of the transition dipole moments of neighboring molecules. This may be particularly prominent for aromatic systems for which the spectra often depend on the type of packing or for which also a wetting layer may have a very different orientation from the following layers. As an excitation of 365 nm was chosen the authors worked close to the onset of the UV-spectrum. Disordering due to structural chemical changes of an initially highly ordered layer may have a strong influence on the absorbance and thus on the photochemical efficiency.

Manuscript NCOMMS-19-03156A
Title: Solar energy storage at an atomically defined organic-oxide hybrid interface
Authors: Christian Schuschke, Chantal Hohner, Martyn Jevric, Anne Ugleholdt Petersen, Zhihang Wang, Matthias Schwarz, Miroslav Kettner, Fabian Waidhas, Lukas Fromm, Christopher Sumby, Andreas Görling, Olaf Brummel, Kasper Moth-Poulsen, and Jörg Libuda

Response of the Authors to the Referee Report and List of Revisions
First, we want to thank the referees for their positive feedback. Below, we address the final remark of Referee 2.

"Referee comments are reproduced in italics"
Replies are given in standard font.
Changes made in the manuscript are highlighted in bold.

Referee 2:

"Most of the questions have been answered to my satisfaction. I only disagree with respect to the statement that UV-Vis spectra are only different in the solid state from the solvent state because of chemical changes due to the anchoring. J-aggregates or H-aggregates exhibit substantially shifted spectra because of Davidov-splitting and modified selection rules because of interactions of the transition dipole moments of neighboring molecules. This may be particularly prominent for aromatic systems for which the spectra often depend on the type of packing or for which also a wetting layer may have a very different orientation from the following layers. As an excitation of 365 nm was chosen the authors worked close to the onset of the UV-spectrum. Disordering due to structural chemical changes of an initially highly ordered layer may have a strong influence on the absorbance and thus on the photochemical efficiency."

We agree to the referee's comment. The intermolecular interaction and orientation effects in the first layer may have an influence on the absorption spectrum. We added an additional remark regarding the role of intermolecular interactions and orientation effects.

Page 10, line 7ff:
Interestingly, the initial photoconversion probability also decreases with decreasing film thickness (see Figure 3e). Experimentally we observe a decrease by a factor of 40 (±50 %) between the 140 ML and the 1 ML film. This effect may be caused by **the preferential molecular orientation in the monolayer, intermolecular interactions which modify the absorption spectrum, and** screening of the electric field of the incident UV light at the metallic Ir substrate.
